# Discovery of a potent olaparib–chlorambucil hybrid inhibitor of PARP1 for the treatment of cancer

**DOI:** 10.3389/fphar.2022.1054616

**Published:** 2023-01-09

**Authors:** Hongyu Qin, Jian Zhang, Yilu Zhao, Lihui Zhang, Jinhong Feng, Lei Zhang

**Affiliations:** ^1^ Department of Medicinal Chemistry, School of Pharmacy, Weifang Medical University, Weifang, Shandong, China; ^2^ School of Stomatology, Weifang Medical University, Weifang, Shandong, China; ^3^ Shandong Analysis and Test Center, Qilu University of Technology (Shandong Academy of sciences), Jinan, Shandong, China

**Keywords:** PARP, olaparib, chlorambucil, cancer, structural modification

## Abstract

**Introduction:** Development of Poly (ADP-ribose) polymerase (PARP) inhibitors has been extensively studied in cancer treatment. Olaparib, the first approved PARP inhibitor, showed potency in the inhibition of both BRCA (breast cancer associated)-mutated and BRCA-unmutated cancers.

**Methods:** Aiming to the discovery of olaparib analogs for the treatment of cancer, structural modifications were performed based on the scaffold of olaparib. In the first series, reduction of carbonyl group to CH_2_ led to decrease of PARP1 inhibitory activity. Preserving the original carbonyl group, molecules with potent PARP1 inhibitory activities were derived by introduction of hydrazide and aromatic nitrogen mustard groups. The synthesized compounds were evaluated in the in the PARP1 enzyme inhibitory screening, cancer cell based antiproliferative assay, cell cycle arrest and apoptosis studies.

**Results:** It is remarkable that, molecule **C2** with chlorambucil substitution, exhibited potent PARP1 inhibitory activity and a broad-spectrum of anticancer potency in the *in vitro* antiproliferative assay. Compared with olaparib and chlorambucil, molecule **C2** also showed significant potency in inhibition of a variety of BRCA-unmutated cell lines. Further analysis revealed the effects of **C2** in induction of G2/M phase cell cycle arrest and promotion of apoptosis.

**Discussion:** Collectively, the olaparib-chlorambucil hybrid molecule **(C2)** could be utilized as a lead compound for further drug design.

## Introduction

Poly (ADP-ribose) polymerases (PARPs) are a group of enzymes with the biological function of transferring ADP-ribose to target proteins (Krishnakumar and Kraus, 2010). PARPs play crucial roles in a variety of cellular processes, including regulation of transcription, replication, recombination, apoptosis, and DNA damage response (Pines et al., 2012; Hu et al., 2014). Within the PARP family, PARP1 serves a critical function in DNA repair and has thus received considerable interest as a major target of inhibitor development (Chaudhuri and Nussenzweig, 2017). For more than a decade, inhibition of PARP1 has been a promising therapeutic intervention strategy for cancer (Coyne et al., 2015).

**FIGURE 1:**
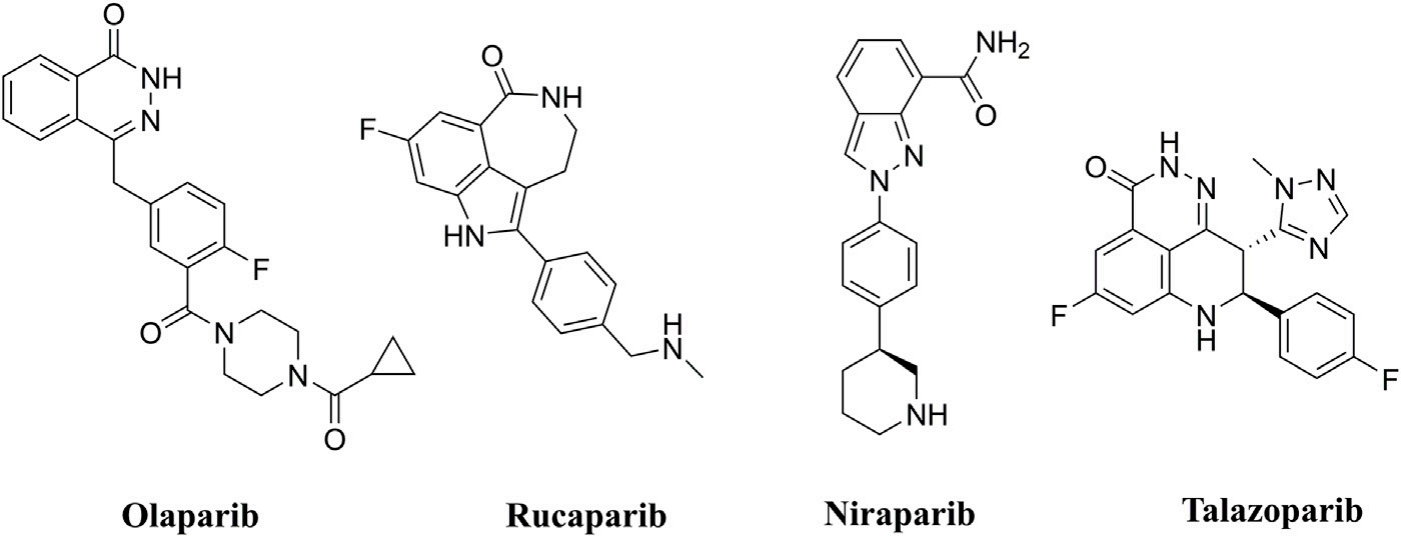
FDA approved PARP inhibitors utilized for the treatment of cancer.

PARP inhibitors (PARPis) have been widely recognized for their significance in inhibition of homologous recombination repair (HR)-deficient tumors (Wang et al., 2016; Rose et al., 2020). In synthetically lethal interactions, tumor cells with essential HR gene mutations in the breast cancer-associated 1 and 2 (BRCA1 and BRCA2) genes can be targeted by PARPis (Bryant et al., 2005). PARPis are the first approved antitumor agents targeting the DNA damage response in BRCA1/2-mutated breast and ovarian cancers (Dockery et al., 2017). Several PARP inhibitors, including olaparib (Menear et al., 2008), rucaparib (Thomas et al., 2007), niraparib (Jones et al., 2015), and talazoparib (Shen et al., 2013), are approved for the treatment of ovarian, breast, and pancreatic cancers with BRCA mutations (Figure 1).

**FIGURE 2:**
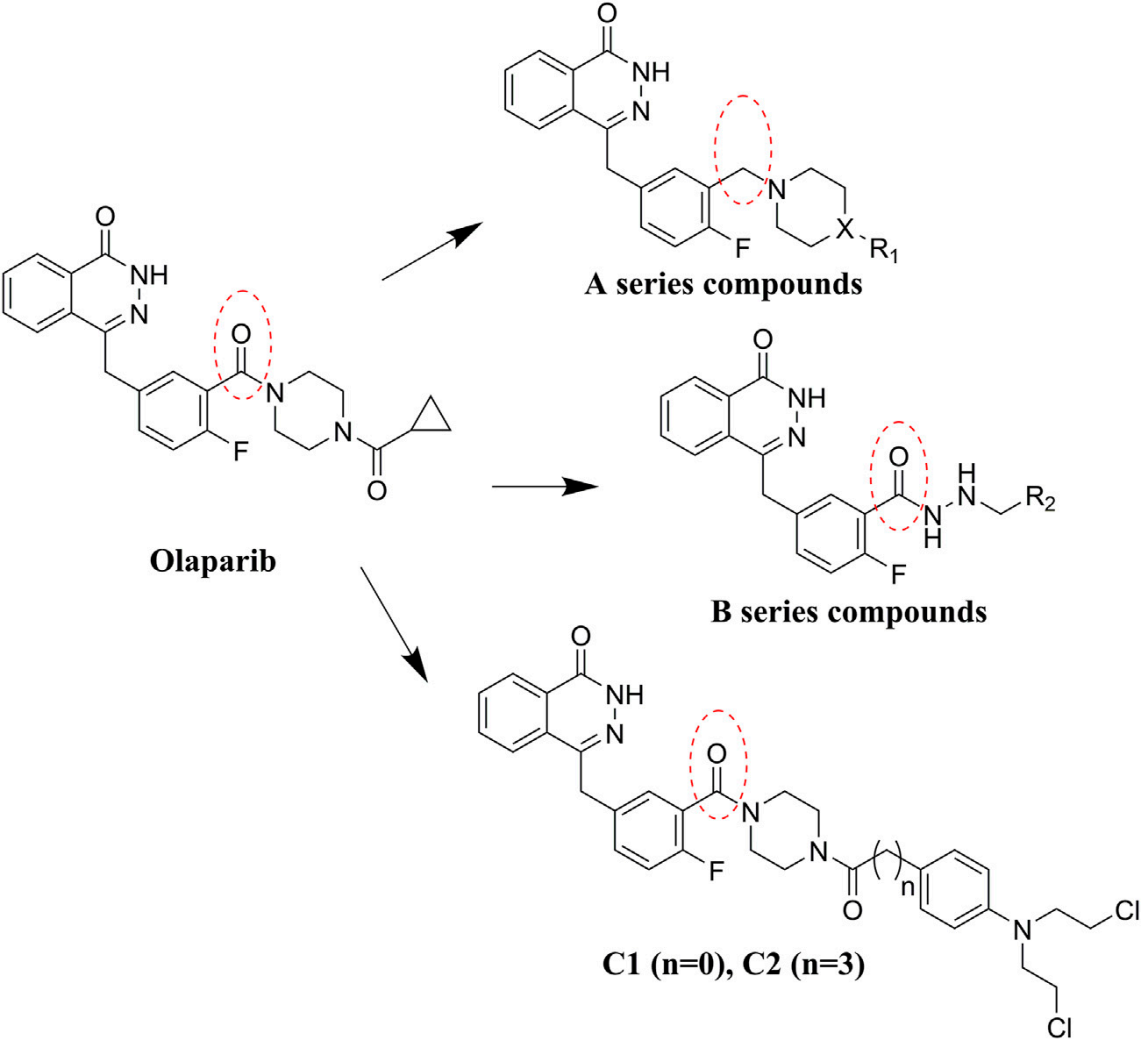
Design of olaparib derivatives in the SAR study.

Olaparib, the first approved small molecule PARPi, is utilized for the treatment of germline BRCA-mutated ovarian and breast cancers. However, there are also some drawbacks that limit the wide application of olaparib, such as its low solubility in both aqueous and lipophilic solutions (Gala et al., 2020). Thus, olaparib was characterized with relatively poor pharmacokinetics profiles and bioavailability (Jackson and Chester, 2015). Therefore, in the search for novel olaparib analogs, we have tested molecules derived from structural modification of olaparib. First, the carbonyl group connected to the piperazine ring was reduced to CH_2_, and a series of olaparib derivatives were synthesized to improve solubility (Figure 2). In further structural derivatization, hydrazide and aromatic nitrogen mustard groups were introduced without reduction of the carbonyl group. The synthesized molecules were evaluated using PARP1 enzyme inhibitory screening and cancer cell-based antiproliferative assays and apoptosis studies.

## Chemistry

**SCHEME 1:**
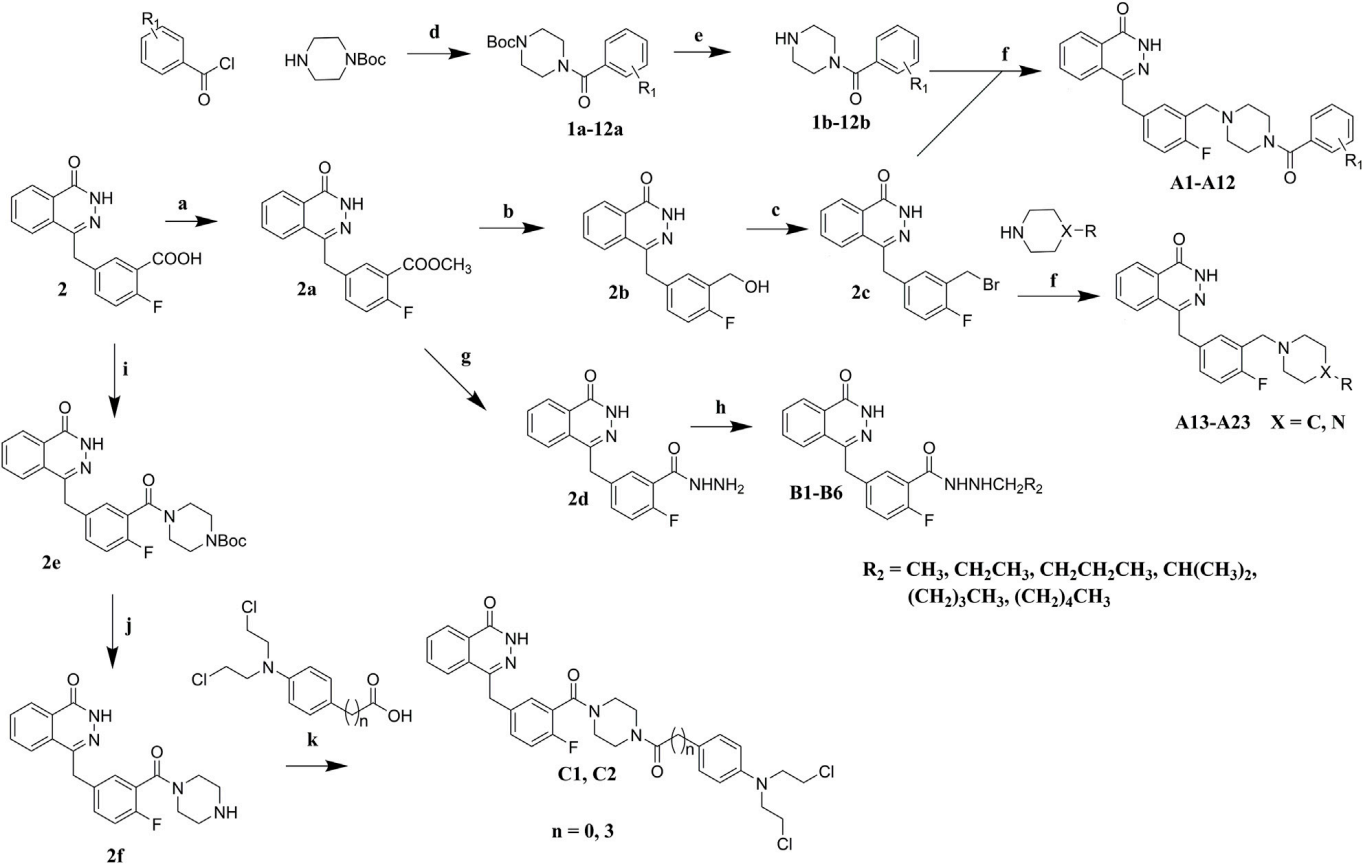
Synthesis of target compounds. Reagents and conditions: (a) H_2_SO_4_, MeOH, reflux; (b) LiAlH_4_, THF, 0°C, RT; (c) PBr_3_, DMF, 0°C; (d) Et_3_N, DCM, 0°C, RT; (e) TFA, DCM, RT; (f) K_2_CO_3_, DCM, RT; (g) NH_2_NH_2_.H_2_O, MeOH, 80°C; (h) R_2_CHO, NaBH_4_, MeOH; (i) Et_3_N, TBTU, Boc-piperazine, DCM, 0°C, RT; (j) TFA, DCM, RT; (k) Et_3_N, TBTU, DCM, 0°C, RT.

Commercially available compound **2** was used as the starting material for the synthesis of the key intermediates in the synthesis (Scheme 1). Intermediates **2a** and **2e** were obtained by esterification and condensation of starting material **2**, respectively. In the synthesis of **A** series target compounds, compound **2a** was treated by LiAlH_4_ reduction and subsequent bromination of the hydroxyl group to generate intermediate **2c**. The other parts (**1b**–**12b**) of the target molecules were synthesized by the coupling of 1-Boc-piperazine with substituted benzoyl chlorides and deprotection of the Boc group. Target compounds **A1**–**A12** were derived by condensation of **2c** with **1b**–**12b**, and compounds **A13**–**A23** were synthesized by reacting **2c** with various substituted piperazines and piperidines. Target compounds **B1**–**B6** were synthesized by hydrazinolysis of intermediate **2a**, followed by condensation reaction with various aliphatic aldehydes and reduction of Schiff base. Preparation of target compounds **C1** and **C2** were accomplished by removal of the Boc group from the structure of intermediate **2e** and subsequent condensation with substituted nitrogen mustards.

## Results and discussion

### PARP1 inhibitory activity assay

In development of olaparib analogs with improved solubility, the carbonyl group in the structure of olaparib was reduced to CH_2_. A series of olaparib derivatives were synthesized with improved water solubility and liposolubility. To evaluate the PARP1 inhibitory activity of the synthesized compounds, the enzyme-based test was performed. As shown in Table 1, all the derived A series compounds exhibited decreased potency compared with olaparib. In this series, cycloalkane formyl substitutions in piperazine showed relatively high inhibitory activity; these included molecules **A21**, **A22**, and **A23**. Among **A1**–**A3**, fluorine substitution in the meta-position of the phenyl ring resulted in improved PARP1 inhibitory activity. The CF_3_ substitution in the benzene ring (**A4**–**A6**) led to decreased activity compared with the fluorine substitution. A methyl group in the ortho-position of the phenyl ring (**A9**) increased inhibitory activity. Compounds with a methoxy group in the phenyl ring (**A11** and **A13**) did not show favorable activity, while the 1,3-dioxole substitution (**A20**) increased inhibitory potency. Replacement of the carbonyl group with a methylene group between piperazine and the phenyl ring (**A15** and **A18**) decreased PARP1 inhibitory potency. The substituted piperidine R groups (**A14** and **A16**–**A19**) exhibited reduced inhibitory potency compared with the substituted piperazine groups.

**TABLE 1:** Structure and PARP1 inhibitory activities of A series PARP inhibitors.

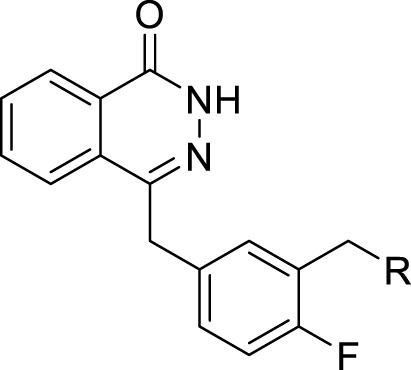		
Compound	R	PARP1 (IC_50_, nM^a^)
A1	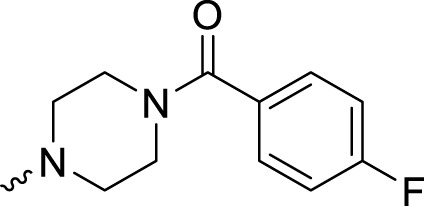	31.2 ± 2.4
A2	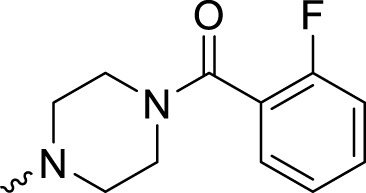	29.4 ± 2.2
A3	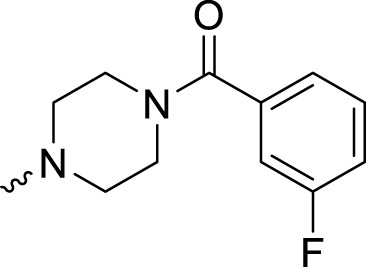	17.6 ± 1.3
A4	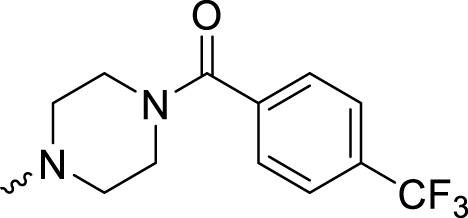	42.2 ± 4.1
A5	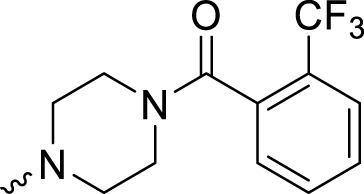	33.2 ± 3.1
A6	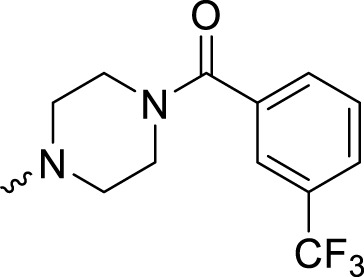	>50
A7	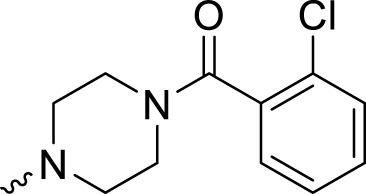	24.6 ± 2.1
A8	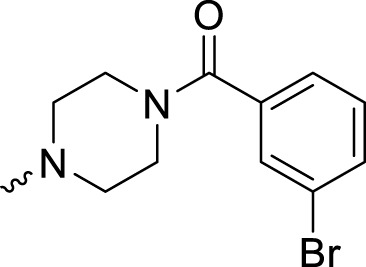	22.5 ± 2.1
A9	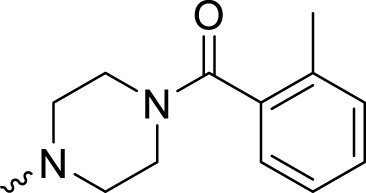	12.6 ± 1.1
A10	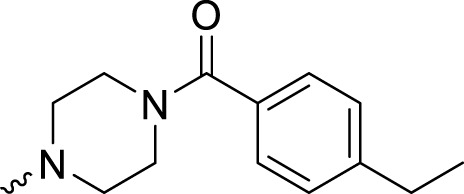	21.4 ± 1.4
A11	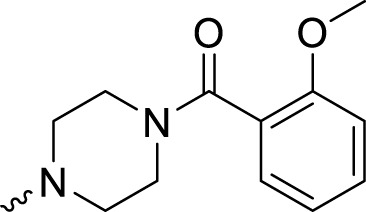	29.4 ± 2.3
A12	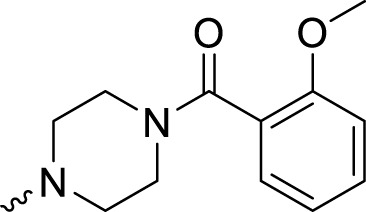	>50
A13	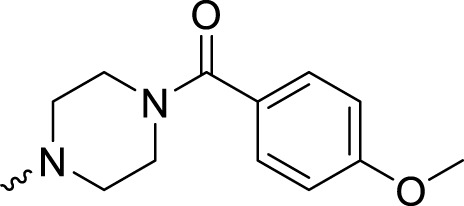	>50
A14	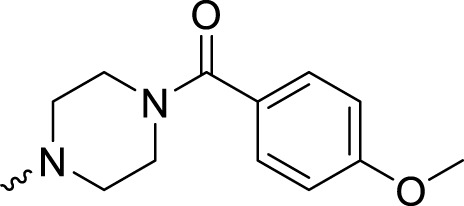	>50
A15	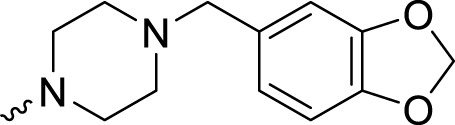	>50
A16	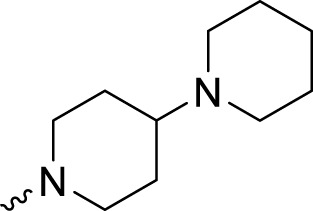	>50
A17	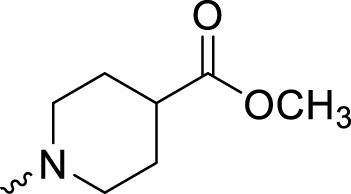	>50
A18	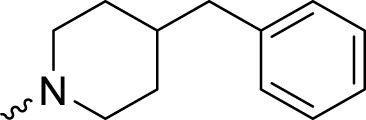	>50
A19	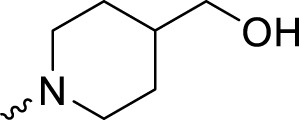	31.5 ± 2.6
A20	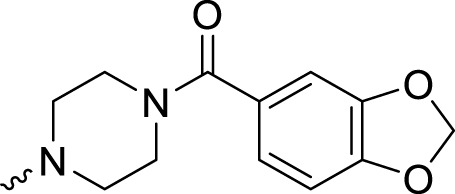	12.6 ± .9
A21	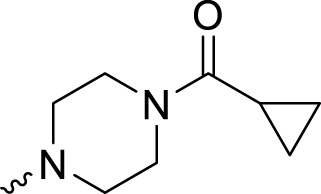	8.4 ± .4
A22	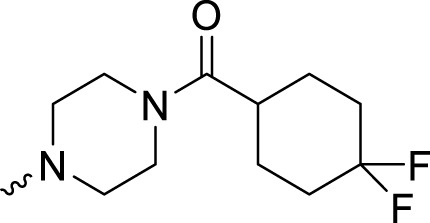	9.3 ± .3
A23	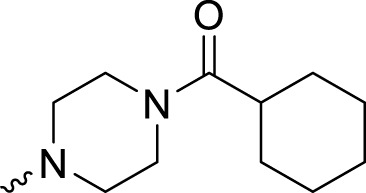	10.1 ± .6
Olaparib		1.2 ± .1

^a^ Each value is the mean of three experiments.

**FIGURE 3:**
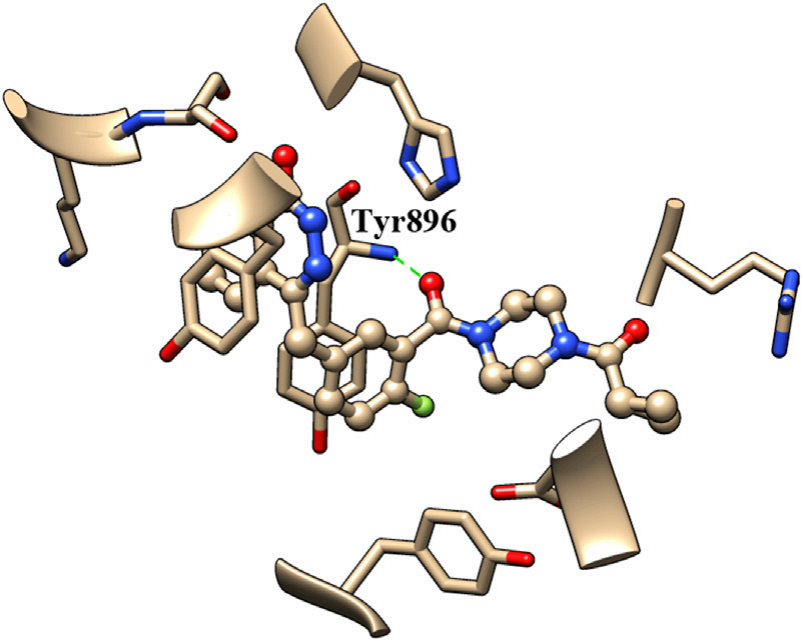
Binding pattern analysis of olaparib in the active site of PARP1.

The binding of inhibitors to the active site of receptors may be key factors that influence inhibitory enzyme potency. Therefore, the crystal structure of olaparib in the active site of PARP1 (PDB entry: 7AAD) was analyzed to identify the structural elements that affect PARP1 inhibitory activity. As shown in Figure 3, the carbonyl group of olaparib could form H-bond interaction with the amino group of Tyr896. The generated H-bond interaction makes an important contribution to the binding of olaparib to the active site of PARP1, with a distance of 2.811 Å between atoms (between the oxygen atom of the carbonyl group and the nitrogen atom of Tyr896). Compared with olaparib, the decreased activity of A series compounds was deduced to be due to the loss of H-bond interaction resulting from the reduction of the carbonyl group.

Considering the importance of the carbonyl group in the structure of olaparib for PARP1 binding, the CO group was retained for further olaparib analog design. Hydrazides with various alkane substitutions were then introduced. The corresponding B series molecules were synthesized, and alkanes of different lengths were utilized to regulate the binding and solubility of target compounds. Nitrogen mustards are known alkylating agents that exert their anticancer activities depending on DNA damage. The aromatic nitrogen mustard groups have been widely used in the design of hybrid anticancer molecules (Chen et al., 2018). It is significant that nitrogen mustards exert their function by damaging DNA, while PARP1 inhibitors prevent DNA repair. Therefore, development of bifunctional alkylating-PARP1 inhibitor molecules may lead to therapeutic agents with synergistic anticancer activities. In this study, to verify the proposed theory, aromatic nitrogen mustard pharmacophores were introduced into the structure of olaparib.

The PARP1 inhibitory activities of **B** series compounds and nitrogen mustard containing **C1** and **C2** were determined using the enzymatic assay (Table 2). Among the **B** series molecules, compound **B3**, with an alkyl chain length of four carbon atoms, exhibited the highest inhibitory activity, with an IC_50_ value of 2.8 nM, compared with olaparib (IC_50_ value of 1.0 nM). Increase or decrease of the alkyl chain length resulted in reduction of PARP1 inhibitory potency. It is also important to note that the compound with branched alkane (**B4**) had reduced activity compared with compounds with straight alkanes. Overall, the **B** series compounds exhibited potent PARP1 inhibitory activity compared with olaparib.

**TABLE 2:** Structure and PARP1 inhibitory activities of B and C series PARP inhibitors.

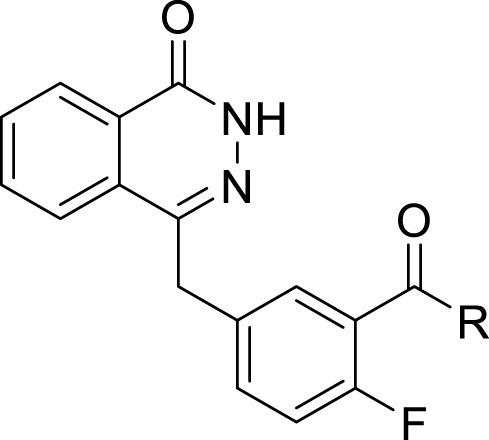		
Compound	R	PARP1 (IC_50_, nM^a^)
B1	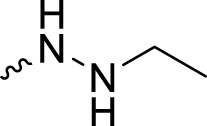	5.1 ± .8
B2	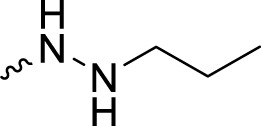	3.8 ± .5
B3	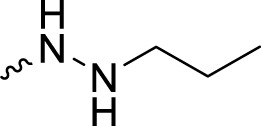	2.8 ± .1
B4	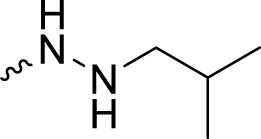	11.8 ± 1.3
B5	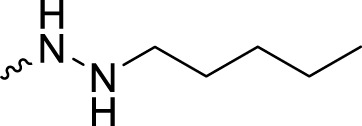	5.2 ± .4
B6	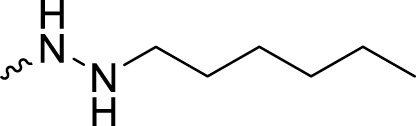	9.4 ± .8
C1	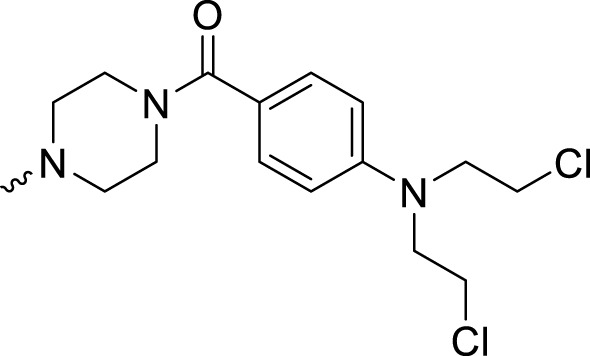	1.2 ± .11
C2	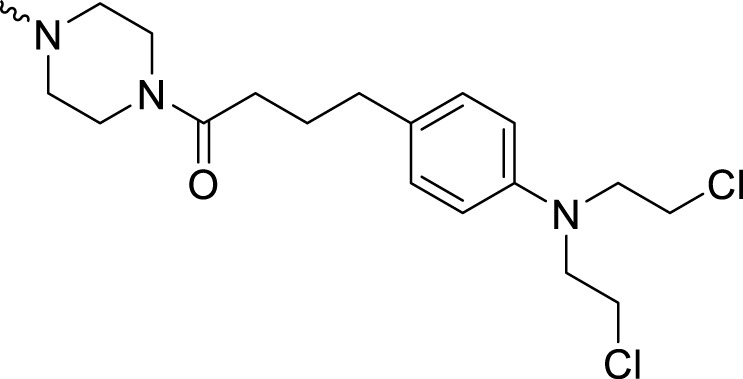	.9 ± .08
Olaparib		1.0 ± .06

^a^ Each value is the mean of three experiments.

**C** series compounds are hybrid molecules with aromatic nitrogen mustard substituted in the piperazine of olaparib. The compound with a long linker (**C2**) exhibited better enzyme inhibitory activity than the compound with a short linker (**C1**). Compared with olaparib (IC_50_ value of 1.0 nM), molecule **C2** had an IC_50_ value of .9 nM in inhibition of PARP1 activity. These data suggest that introduction of an aromatic nitrogen mustard group improves PARP1 inhibitory activity and that the olaparib–chlorambucil hybrid molecule **C2** could be utilized for further evaluation as a potent PARP1 inhibitor.

### Antiproliferative activity test

It has been reported that olaparib has potential efficacy in tumors lacking DNA repair defects (Gelmon et al., 2011; Poveda et al., 2019). The existence of non-DNA repair functions of PARP1 provides targetable opportunities for anticancer drug development (Hanahan and Weinberg, 2011; Weaver and Yang, 2013). Therefore, both BRCA-mutated cell lines (MDA-MB-436, Capan-1, and HCC 1937) and cell lines without BRCA mutations (K562, Raji, 8226, U87, B16, SKOV3, MCF-7, SW620, A549, MDA-MB-231, CH151, U251, and Hela) were utilized for the antiproliferative assay. Olaparib and the alkylating agent chlorambucil were used as the positive control. Only molecules with high PARP1 inhibitory activities (**B3**, **C1**, and **C2**) were selected for the cancer cell-based assay. As shown in Table 3, compound **B3** exhibited very limited antiproliferative activity against the tested cell lines compared with olaparib. These data suggest that the performed hydrazide substitutions could not improve the potency of olaparib in both enzymatic and antiproliferative tests. In inhibition of the BRCA-mutated cell lines (MDA-MB-436, Capan-1, and HCC 1937), molecule **C2** showed activity similar to olaparib. Compared with the positive control, molecule **C2** exhibited high potency in inhibition of several BRCA-unmutated cell lines, such as K562, Raji, U87, MCF-7, A549, CH151, U251, and Hela cells. It was deduced that the chlorambucil part of **C2** contributed to the antiproliferative activity against BRCA-unmutated cells. It was interesting that molecule **C1** selectively inhibited the growth of A549 cells without obvious inhibitory activity against other BRCA-unmutated cells. These data suggest that molecule **C1** could be used as a lead compound for the design of anticancer drugs for the treatment of lung cancer. In summary, molecule **C2** exhibited high anticancer activity *in vitro* in the antiproliferative test, and the olaparib–nitrogen mustard hybridization strategy demonstrated good potential as anticancer therapy.

**TABLE 3:** *In vitro* antiproliferative activities of representative compounds (IC_50_, μM^a^).

	B3	C1	C2	Olaparib	Chlorambucil
MDA-MB-436	>1	.018 ± .002	.013 ± .001	.047 ± .003	>1
Capan-1	>1	.105 ± .010	.086 ± .006	.129 ± .011	>1
HCC1937	>1	.36 ± .02	.18 ± .01	.24 ± .01	>1
K562	>20	>20	1.12 ± .08	10.4 ± 0.72	>20
Raji	>100	>100	1.21 ± .02	4.40 ± .33	40.6 ± 3.6
8226	>100	>100	3.62 ± .20	66.8 ± 5.5	>100
U87	>100	>100	2.86 ± .01	26.0 ± 1.2	>100
B16	>100	>100	11.7 ± 1.01	>100	>100
SKOV3	>100	>100	4.04 ± .25	12.8 ± 1.2	3.98 ± .22
MCF-7	>100	>100	2.28 ± .11	30.6 ± 2.1	19.5 ± 1.7
SW620	>100	>100	6.41 ± .46	17.7 ± 1.2	10.0 ± 1.01
A549	>100	14.1 ± 1.1	2.01 ± .13	1.62 ± .11	89.4 ± 3.4
MDA-MB-231	>100	>100	6.33 ± .35	53.8 ± 3.6	95.3 ± 5.7
CH151	>100	>100	2.77 ± .13	24.9 ± 1.5	>100
U251	>100	>100	2.58 ± .14	>100	21.1 ± 1.8
Hela	>100	>100	1.65 ± .11	11.6 ± 1.01	>100

^a^Each value is the mean of three experiments.

### Cell cycle analysis

**FIGURE 4:**
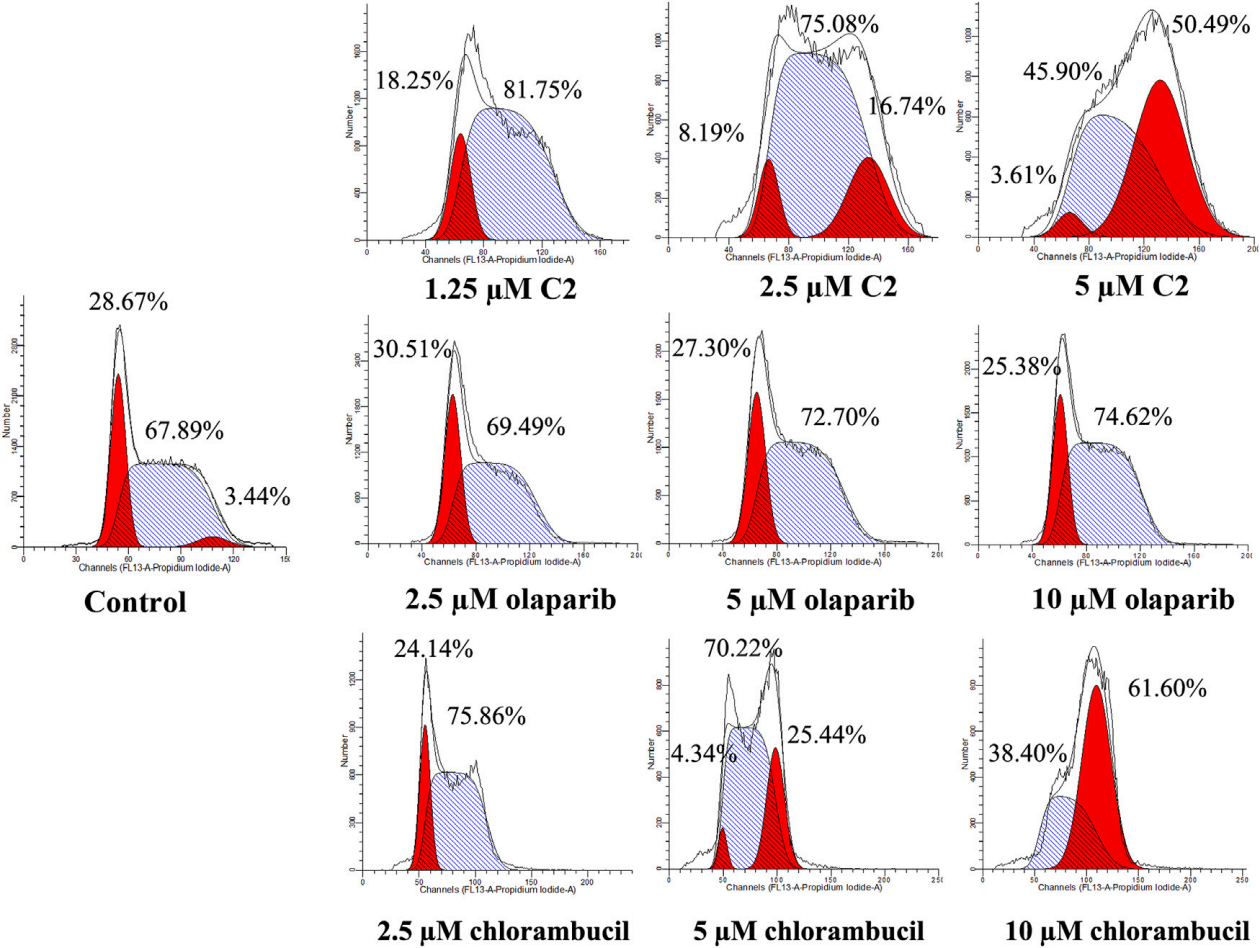
Cell cycle analysis of K562 cells treated with molecule **C2**, olaparib, or chlorambucil. Cells were treated with molecule **C2** (1.25, 2.5, or 5.0 µM), olaparib, or chlorambucil (2.5, 5.0, or 10 µM) at different concentrations for 24 h. The results were evaluated using flow cytometry.

**FIGURE 5:**
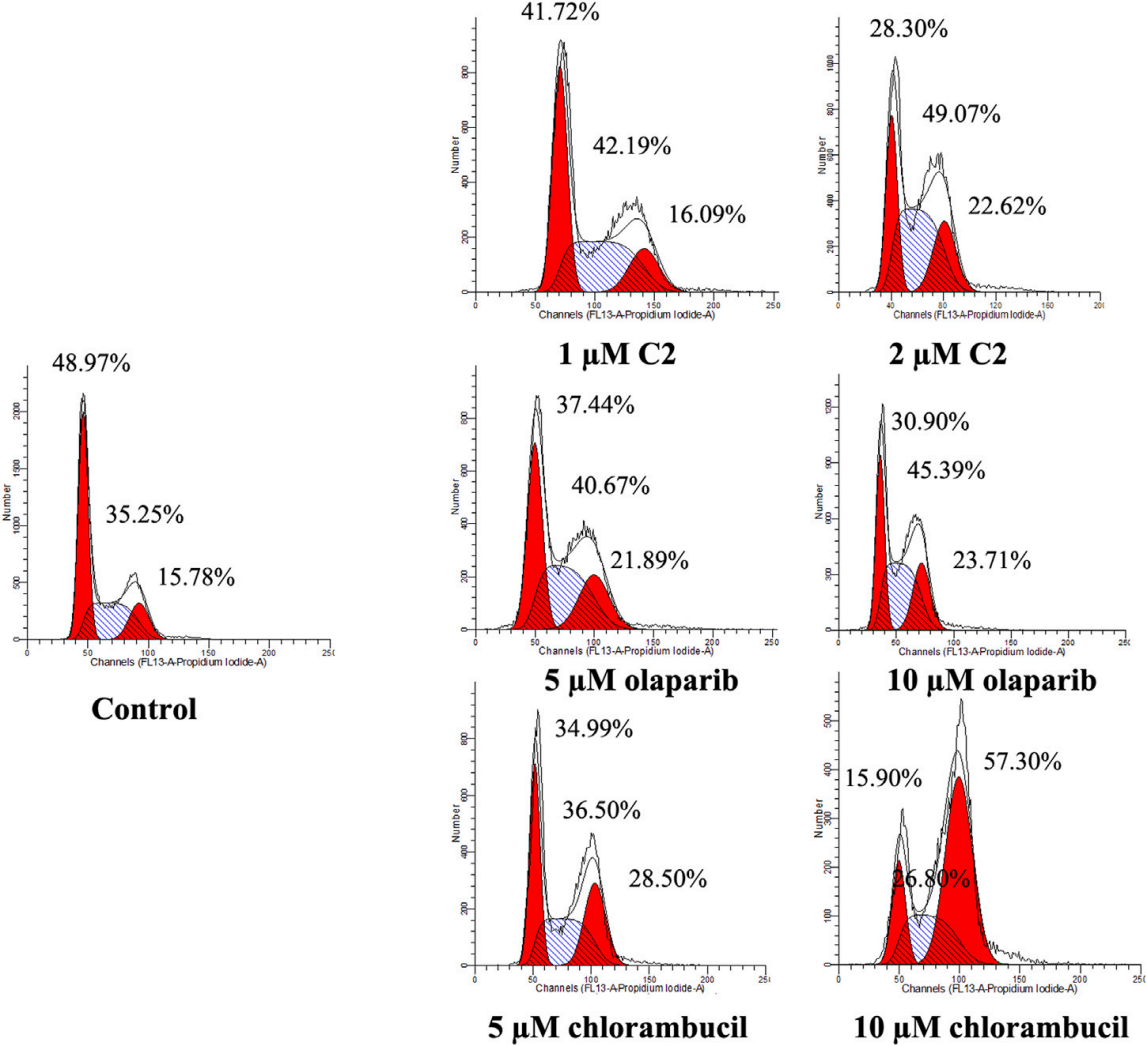
Cell cycle analysis of HCC1937 cells treated with molecule **C2**, olaparib, or chlorambucil. Cells were treated with molecule **C2** (1 or 2 µM), olaparib, or chlorambucil (5 or 10 µM) at different concentrations for 24 h. The results were evaluated using flow cytometry.

A characteristic change in tumor cells is dysregulation of the cell cycle due to genetic mutations, resulting in uncontrolled cell proliferation. Cell cycle arrest is usually induced by chemotherapeutic drugs in cancer treatment. Therefore, in this study, the effect of **C2** on cell cycle progression was evaluated in K562 cells at doses of 1.25–5.0 µM. As shown in Figure 4, **C2** and chlorambucil increased cell number at the G2/M phase in a dose-dependent manner. The proportion of cells in the G2/M phase increased from 0% to 45.9% with **C2** treatment (corresponding to concentration increase from 1.25 to 5.0 µM); with chlorambucil treatment, cells at the G2/M phase increased from 0% to 61.60% when concentration was increased from 2.5 to 10 µM. However, at the tested concentrations of 2.5–10 µM, olaparib did not exhibit any effects on regulation of cell cycle in K562 cells. The BRCA1-mutated HCC1937 human breast cancer cell line was also utilized to evaluate the cell cycle arrest patterns induced by molecule **C2**, olaparib, and chlorambucil. Figure 5 reveals that all the tested compounds increased cell proportion in the G2/M phase. These results suggest that induction of G2/M phase arrest plays an important role in the *in vitro* anticancer effects of molecule **C2**.

### Analysis of apoptosis

**FIGURE 6:**
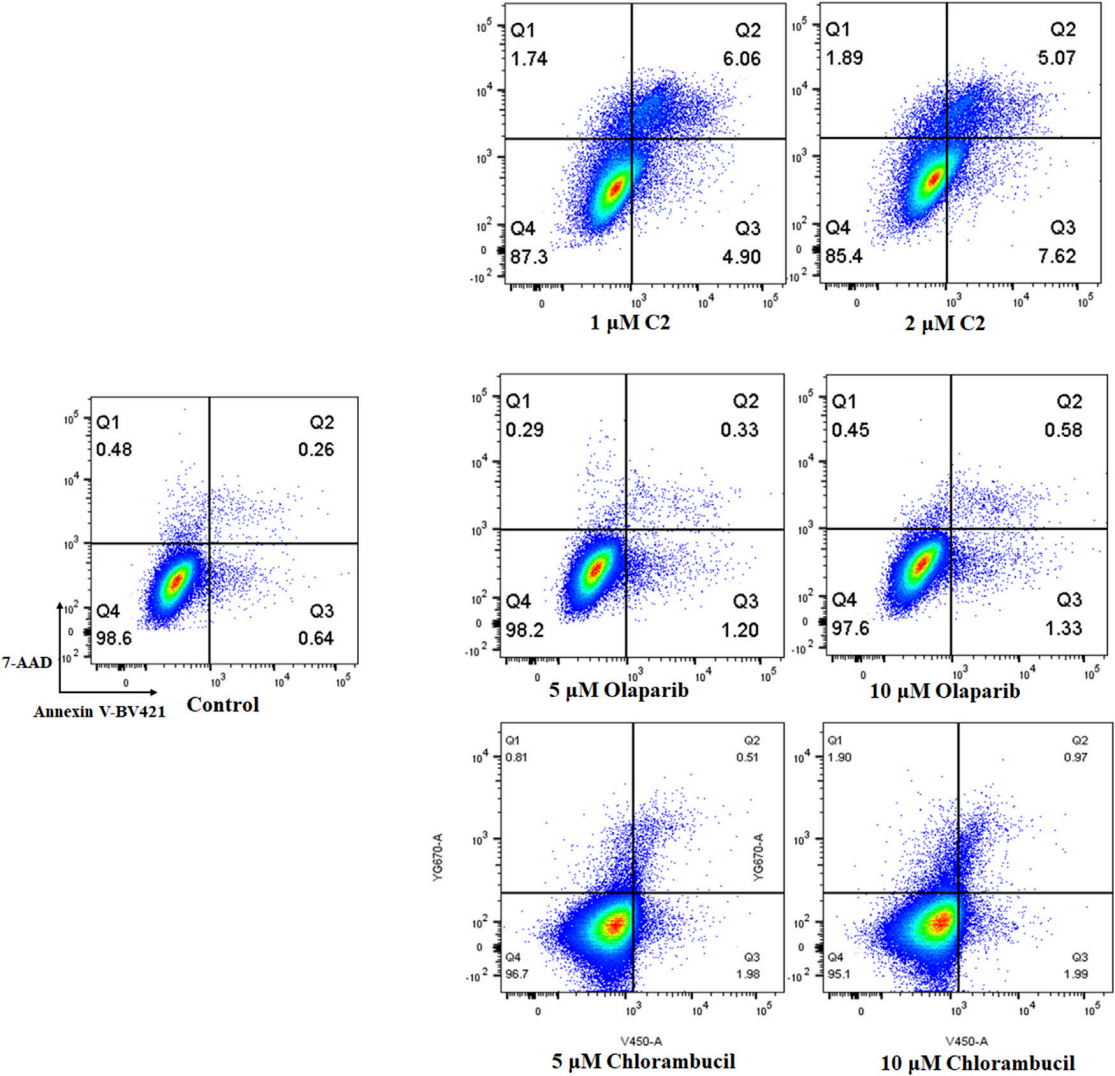
Proapoptotic effects of molecule **C2**. K562 cells were treated with molecule **C2** (1 or 2 µM), olaparib (5 or 10 µM), or chlorambucil (5 or 10 µM) for 24 h. The cells were then stained with Annexin V–BV421/7-AAD, and the apoptotic status of the cells was assessed using flow cytometry.

Apoptosis plays an important role in the inhibition of cancer. PARP inhibitors have been used to induce apoptosis in different cancer cell lines. Therefore, the effect of molecule **C2** in the induction of cancer cell apoptosis was evaluated. K562 cells, which were sensitive to the test compounds, were selected for the apoptosis study and analyzed by annexin V staining. The results demonstrated that an increased number of apoptotic K562 cells were detected after treatment with different doses of **C2**, olaparib, or chlorambucil (Figure 6). It is noteworthy that molecule **C2** induced K562 cell apoptosis at higher rates with lower doses than olaparib and chlorambucil. The percentage of apoptotic cells was significantly increased from .90% in the control to 10.96% and 12.69% after treatment with 1 μM and 2 µM of **C2**, respectively, compared with olaparib (apoptotic rates of 1.53% and 1.91% at doses of 5 μM and 10 µM, respectively) and chlorambucil (apoptotic rates of 2.49% and 2.96% at doses of 5 μM and 10 µM, respectively). These data suggest that induction of apoptosis plays an important role in the anticancer effects of molecule **C2**.

## Conclusion

Inhibition of PARPs has emerged as a research focus in the development of targeted cancer treatments. Four PARP inhibitors are currently approved for clinical use: olaparib, rucaparib, niraparib, and talazoparib. As the first approved PARP inhibitor, olaparib exhibited potency in the inhibition of both BRCA-mutated and BRCA-unmutated cancers. In development of novel PARP inhibitors as anticancer drugs, modifications have been made to the structure of olaparib. In the current study, 31 compounds were designed, synthesized, and tested for activity. Among the derived molecules, an olaparib–chlorambucil hybrid molecule (**C2**) exhibited high potency in the inhibition of PARP1 activity and showed high antiproliferative activity against a group of cancer lines. Notably, in the inhibition of multiple BRCA-unmutated cancer cells, molecule **C2** showed improved anticancer activity *in vitro* compared with olaparib and chlorambucil. Analysis of the cell cycle and apoptosis showed that G2/M phase cell cycle arrest and apoptosis are involved in the anticancer effects of **C2**. Our study led to the discovery of a potent PARP1 inhibitor–chlorambucil hybrid compound with strong anticancer effects. Our results also revealed the potential of PARP inhibitor–chemotherapeutic drug hybrids in the treatment of cancer.

## Materials and methods

All commercially available materials, reagents, and solvents were used without further purification. All reactions were monitored using TLC with .25-mm silica gel plates (60GF-254). UV light and ferric chloride were used to visualize the spots. ^1^H NMR and ^13^C NMR spectra were recorded on a Bruker DRX spectrometer at 500 MHz, using TMS as an internal standard. High-resolution mass spectra were produced at Weifang Medical University.

### Preparation of **1a** and its analogs: Derivatives **2a–12a** were prepared as described for **1a** (see as follows)

*Tert*-butyl 4-(4-fluorobenzoyl)piperazine-1-carboxylate (**1a**). To a solution of 1-Boc-piperazine (1.86 g, 10 mmol) in DCM, the following was added dropwise: 4-fluorobenzoyl chloride (1.90 g, 12 mmol), followed by Et_3_N (3.03 g, 30 mmol) at 0°C; the mixture was stirred for 4 h. The solvent was then evaporated, and the residue was taken up in EtOAc (50 ml). The EtOAc solution was washed with saturated citric acid (3 × 50 ml), NaHCO_3_ (3 × 50 ml), and saturated brine solution (3 × 50 ml), dried over MgSO_4_, and evaporated under a vacuum. The crude product was used directly in the next step.

### Preparation of **1b** and its analogs: Derivatives **2b–12b** were prepared as described for **1b** (see as follows)

(4-Fluorophenyl)(piperazin-1-yl)methanone (**1b**). To a solution of **1a** in DCM with TFA at RT, and the mixture was stirred for 2 h. The mixture was then concentrated *in vacuo*. The crude oil was used for the following step without purification.

### Preparation of **2a** (see as follows)

Methyl 2-fluoro-5-((4-oxo-3,4-dihydrophthalazin-1-yl)methyl)benzoate (**2a**). Three drops of concentrated sulfuric acid were added to the methanol solution of dissolved compound **2** (2.98 g, 10 mmol). The mixture was heated at reflux for 5 h in an oil bath. TLC analysis indicated the complete disappearance of the starting material. Upon cooling to ambient temperature, the solvent was evaporated under vacuum. The residue was extracted with EtOAc (50 ml) and saturated NaHCO_3_ (3 × 50 ml), dried over MgSO_4_, and evaporated under vacuum. The desired compound **2a** (2.97 g, 95% yield) was derived as white powder. HRMS m/z: 313.09586 [M + H]^+^. ^1^H-NMR (400 MHz, DMSO) δ 12.62 (s, 1H), 8.27 (d, *J* = 8.0 Hz, 1H), 8.00 (d, *J* = 8.0 Hz, 1H), 7.93–7.83 (m, 3H), 7.63 (s, 1H), 7.30 (t, *J* = 8.0 Hz, 1H), 4.39 (s, 2H), and 3.84 (s, 3H).

### Preparation of **2b** (see as follows)

(5-Fluoro-3-(hydroxymethyl)benzyl)phthalazin-1(2*H*)-one (**2b**). A solution of LiAlH_4_ (.12 g, 3.20 mmol) in THF was stirred at 0°C; after 10 min, compound **2a** (.50 g, 1.60 mmol) was added. The reaction solution was stirred at room temperature for 30 min. Then ice water was poured into the reaction mixture, and the solution was adjusted to pH 5-6 using 1 M HCl. The target compound **2b** (.36 g, 80% yield) was obtained by filtering. HRMS m/z: 283.08902 [M + H]^+^. ^1^H-NMR (400 MHz, DMSO) δ 12.59 (s, 1H), 8.26 (q, *J* = 4.0 Hz, 1H), 7.94 (d, *J* = 8.0 Hz, 1H), 7.89–7.79 (m, 2H), 7.40–7.38 (m, 1H), 7.28–7.24 (m, 1H), 7.08–7.04 (m, 1H), 5.23–5.20 (m, 1H), 4.48 (d, *J* = 8.0 Hz, 2H), and 4.29 (s, 2H).

### Preparation of **2c** (see as follows)

4-(3-(Bromomethyl)-4-fluorobenzyl)phthalazin-1(2*H*)-one (**2c**). The starting material, **2b** (.20 g, .71 mmol), was dissolved in DMF, and the solution was cooled to 0°C. PBr_3_ (.29 g, 1.07 mmol) was added to the solution at 0°C, and then, the mixture was stirred for 30 min. The reaction was quenched by addition of H_2_O and extracted with EtOAc. The combined organic layers were dried over Na_2_SO_4_, filtered, and evaporated under vacuum. The target compound **2c** (.18 g, 74% yield) was derived by crystallization in ether. HRMS m/z: 347.01743 [M + H]^+^. ^1^H-NMR (400 MHz, DMSO) δ 12.59 (s, 1H), 8.26 (d, *J* = 8.0 Hz, 1H), 7.95–7.90 (m, 1H), 7.85 (dd, *J* = 8.0, 1.2 Hz, 1H), 7.81 (dd, *J* = 6.0, 1.2 Hz, 1H), 7.45 (dd, *J* = 8.0, 2.0 Hz, 1H), 7.36 (dd, *J* = 2.8, 2.4 Hz, 1H), 7.34–7.33 (m, 1H), 7.19–7.14 (m, 1H), 4.64 (s, 2H), and 4.29 (s, 2H).

### Preparation of **A1** and its analogs: Derivatives **A2–A23** were prepared as described for **A1** (see as follows)

4-(4-Fluoro-3-((4-(4-fluorobenzoyl)piperazin-1-yl)methyl)benzyl)phthalazin-1(2*H*)-one (**A1**). To a solution of **2c** (.15 g, .43 mmol) and **1b** (.10 g, .48 mmol) in DCM, K_2_CO_3_ (.18 g, 1.29 mmol) was added at RT, and the mixture was stirred overnight. The solvent was evaporated, and the residue was dissolved in EtOAc (20 ml). The EtOAc solution was then washed with saturated brine (3 × 20 ml), dried over MgSO_4_, and evaporated under vacuum. The desired compound was derived by crystallization in EtOAc as white powder. HRMS (AP-ESI) m/z calcd for C_27_H_24_F_2_N_4_O_2_ [M + H]^+^ 475.19473, found 475.19174. ^1^H-NMR (400 MHz, DMSO) δ 12.59 (s, 1H), 8.25 (d, *J* = 8.0 Hz, 1H), 7.91 (d, *J* = 8.0 Hz, 1H), 7.88–7.77 (m, 2H), 7.46–7.42 (m, 2H), 7.33–7.24 (m, 4H), 7.11–7.07 (m, 1H), 4.30 (s, 2H), 3.50–3.16 (m, 6H), and 2.33 (s, 2H). ^13^C-NMR (400 MHz, DMSO) δ 168.451, 164.208, 161.756, 161.164, 159.870, 145.629, 134.439, 133.838, 132.655 (d, *J* = 74.2 Hz), 130.042 (d, *J* = 8.5 Hz), 129.655, 128.364, 126.486, 126.088, 124.516, 115.954, 65.385, 54.677, 52.773, 40.398 (d, *J* = 21.0 Hz), 39.980, and 37.257 ppm.

4-(4-Fluoro-3-((4-(2-fluorobenzoyl)piperazin-1-yl)methyl)benzyl)phthalazin-1(2*H*)-one (**A2**). HRMS (AP-ESI) m/z calcd for C_27_H_24_F_2_N_4_O_2_ [M + H]^+^ 475.19473, found 475.19235 [M + H]^+^. ^1^H-NMR (400 MHz, DMSO) δ 12.59 (s, 1H), 8.25–8.24 (m, 1H), 7.91 (d, *J* = 8.0 Hz, 1H), 7.86–7.77 (m, 2H), 7.51–7.49 (m, 2H), 7.38–7.24 (m, 4H), 7.11–7.07 (m, 1H), 4.30 (s, 2H), 3.58 (s, 2H), 3.50 (s, 2H), 3.15 (s, 2H), 2.37 (s, 2H), and 2.28–2.26 (m, 2H). ^13^C-NMR (400 MHz, DMSO) δ 164.273, 161.164, 159.862, 159.168, 156.729, 145.622, 134.472, 133.829, 132.064, 131.909, 131.871 (d, *J* = 11.2 Hz), 129.278, 128.365, 126.483, 126.099, 125.423, 124.569, 116.353, 115.835, 54.669, 52.903, 52.337, 46.926, 41.682, and 37.247 ppm.

4-(4-Fluoro-3-((4-(3-fluorobenzoyl)piperazin-1-yl)methyl)benzyl)phthalazin-1(2*H*)-one (**A3**). HRMS (AP-ESI) m/z calcd for C_27_H_24_F_2_N_4_O_2_ [M + H]^+^ 475.19473, found 475.19229 [M + H]^+^. ^1^H-NMR (400 MHz, DMSO) δ 12.58 (s, 1H), 8.26–8.23 (m, 1H), 7.92–7.90 (m, 1H), 7.87–7.76 (m, 2H), 7.53–7.47 (m, 1H), 7.33–7.19 (m, 5H), 7.11–7.07 (m, 1H), 4.29 (s, 2H), 3.54 (s, 2H), 3.50 (s, 2H), 3.24 (s, 2H), 2.36 (s, 2H), and 2.23 (s, 2H). ^13^C-NMR (400 MHz, DMSO) δ 167.851 (d, *J* = 2.1 Hz), 163.500, 161.164 (d, *J* = 10.3 Hz), 161.062, 159.871, 145.612, 138.656 (d, *J* = 6.8 Hz), 134.458 (d, *J* = 3.1 Hz), 133.816, 132.016, 131.885, 131.122 (d, *J* = 8.1 Hz), 129.733, 128.370, 126.077, 124.370 (d, *J* = 15.1 Hz), 123.402, 116.945, 115.604, 114.501, 54.677, 52.846, 52.306, 47.441, 41.906, and 37.263 ppm.

4-(4-Fluoro-3-((4-(4-(trifluoromethyl)benzoyl)piperazin-1-yl)methyl)benzyl)phthalazin-1(2*H*)-one (**A4**). HRMS (AP-ESI) m/z calcd for C_28_H_24_F_4_N_4_O_2_ [M + H]^+^ 525.19154, found 525.18939 [M + H]^+^. ^1^H-NMR (400 MHz, DMSO) δ 12.59 (s, 1H), 8.25 (dd, *J*_1_ = 6.8 Hz, *J*_2_ = 1.2 Hz, 1H), 7.92 (d, *J* = 8.0 Hz, 1H), 7.87–7.77 (m, 4H), 7.59 (d, *J* = 8.0 Hz, 2H), 7.33–7.24 (m, 2H), 7.12–7.07 (m, 1H), 4.30 (s, 2H), 3.58 (s, 2H), 3.51 (s, 2H), 3.22 (s, 2H), 2.39 (s, 2H), and 2.30 (s, 2H). ^13^C-NMR (400 MHz, DMSO) δ 167.966, 161.170, 159.869, 158.751, 145.620, 140.434, 134.465 (d, *J* = 3.1 Hz), 133.839, 132.044, 131.899, 130.248, 129.681, 128.187 (d, *J* = 17.9 Hz), 126.479, 125.977, 124.348 (d, *J* = 15.1 Hz), 123.043, 115.828, 115.607, 54.669, 52.857, 52.277, 47.434, 41.890, and 37.252 ppm.

4-(4-Fluoro-3-((4-(2-(trifluoromethyl)benzoyl)piperazin-1-yl)methyl)benzyl)phthalazin-1(2*H*)-one (**A5**). HRMS (AP-ESI) m/z calcd for C_28_H_24_F_4_N_4_O_2_ [M + H]^+^ 525.19154, found 525.18878 [M + H]^+^. ^1^H-NMR (400 MHz, DMSO) δ 12.59 (s, 1H), 8.24 (dd, *J*_1_ = 8.0 Hz, *J*_2_ = 2.8 Hz, 1H), 7.91 (d, *J* = 8.0 Hz, 1H), 7.86–7.73 (m, 4H), 7.66 (d, *J* = 8.0 Hz, 1H), 7.44–7.42 (m, 1H), 7.33–7.31 (m, 1H), 7.25–7.23 (m, 1H), 7.11–7.06 (m, 1H), 4.29 (s, 2H), 3.57 (s, 2H), 3.49 (s, 2H), 3.05–2.97 (m, 2H), and 2.41–2.19 (m, 4H). ^13^C-NMR (400 MHz, DMSO) δ 166.462, 161.155, 159.869, 159.735, 145.596, 135.171, 134.462, 133.329, 132.006, 131.967, 129.923, 129.651 (d, *J* = 8.6 Hz), 128.363, 127.009, 126.060, 125.536, 124.359, 122.836, 115.808, 54.685, 52.360, 52.161, 46.989, 41.464, and 37.250 ppm.

4-(4-Fluoro-3-((4-(3-(trifluoromethyl)benzoyl)piperazin-1-yl)methyl)benzyl)phthalazin-1(2*H*)-one (**A6**). HRMS (AP-ESI) m/z calcd for C_28_H_24_F_4_N_4_O_2_ [M + H]^+^ 525.19154, found 525.18993 [M + H]^+^. ^1^H-NMR (400 MHz, DMSO) δ 12.59 (s, 1H), 8.24 (q, *J* = 4.0 Hz, 1H), 7.91 (*J* = dd, *J*_1_ = 8.0 Hz, *J*_2_ = 2.8 Hz, 1H), 7.86–7.76 (m, 3H), 7.72–7.68 (m, 2H), 7.34–7.23 (m, 2H), 7.09 (m, 1H), 4.30 (s, 2H), 3.57 (s, 2H), 3.50 (s, 2H), 3.24 (s, 2H), 2.39 (s, 2H), and 2.30 (s, 2H). ^13^C-NMR (400 MHz, DMSO) δ 168.096, 161.647, 159.928, 159.190, 145.215, 136.444, 135.119, 134.753, 134.024, 132.867, 131.938, 131.562, 130.244, 129.486 (d, *J* = 12.6 Hz), 128.378, 127.076, 126.246, 125.693, 124.393, 122.984, 116.198, 52.306, 50.519, and 37.383 ppm.

4-(3-((4-(2-Chlorobenzoyl)piperazin-1-yl)methyl)-4-fluorobenzyl)phthalazin-1(2*H*)-one (**A7**). HRMS (AP-ESI) m/z calcd for C_27_H_24_ClFN_4_O_2_ [M + H]^+^ 491.16518, found 491.16260 [M + H]^+^. ^1^H-NMR (400 MHz, DMSO) δ 12.58 (s, 1H), 8.25 (q, *J* = 4.0 Hz, 1H), 7.91 (d, *J* = 8.0 Hz, 1H), 7.86–7.76 (m, 2H), 7.53 (d, *J* = 8.0 Hz, 1H), 7.47–7.40 (m, 2H), 7.40–7.31 (m, 2H), 7.27–7.23 (m, 1H), 7.11–7.06 (m, 1H), 4.29 (s, 2H), 3.58 (s, 2H), 3.49 (s, 2H), 3.06 (s, 2H), 2.38 (s, 2H), and 2.30 (s, 2H). ^13^C-NMR (400 MHz, DMSO) δ 165.874, 161.156, 159.863, 158.733, 145.617, 136.162, 134.472, 133.839, 131.912, 130.963, 129.877, 129.559, 128.361, 128.062, 126.490, 126.096, 124.339, 115.609 (d, *J* = 22.3 Hz), 54.688, 52.817, 52.264, 46.615, 41.425, and 37.246 ppm.

4-(3-((4-(3-Bromobenzoyl)piperazin-1-yl)methyl)-4-fluorobenzyl)phthalazin-1(2*H*)-one (**A8**). HRMS (AP-ESI) m/z calcd for C_27_H_24_BrFN_4_O_2_ [M + H]^+^ 535.11467, found 535.11224 [M + H]^+^. ^1^H-NMR (400 MHz, DMSO) δ 12.60 (s, 1H), 8.25 (dd, *J*_1_ = 8.0 Hz, *J*_2_ = 1.2 Hz, 1H), 7.92 (d, *J* = 8.0 Hz, 1H), 7.87–7.81 (m, 2H), 7.79–7.66 (m, 1H), 7.56 (s, 1H), 7.44–7.25 (m, 5H), 4.30 (s, 3H), 3.50 (s, 4H), 3.24 (s, 2H), and 2.31 (s, 3H). ^13^C-NMR (400 MHz, DMSO) δ 167.670, 159.889, 134.458, 133.887, 131.929, 131.189, 130.035, 129.546, 128.372, 126.491 (d, *J* = 14.5 Hz), 126.347, 126.129, 122.195, 52.811, 40.600, 40.392, 39.975, 39.766, and 37.285 ppm.

4-(4-Fluoro-3-((4-(2-methylbenzoyl)piperazin-1-yl)methyl)benzyl)phthalazin-1(2*H*)-one (**A9**). HRMS (AP-ESI) m/z calcd for C_28_H_27_FN_4_O_2_ [M + H]^+^ 471.21980, found 471.21762 [M + H]^+^. ^1^H-NMR (400 MHz, DMSO) δ 12.58 (s, 1H), 8.24 (dd, *J*_1_ = 8.0 Hz, *J*_2_ = 1.2 Hz, 1H), 7.91–7.90 (m, 1H), 7.85–7.75 (m, 2H), 7.32–7.21 (m, 5H), 7.12–7.06 (m, 2H), 4.29 (s, 2H), 3.58 (s, 2H), 3.49 (s, 2H), 3.04 (t, *J* = 4.8 Hz, 2H), 2.37 (s, 2H), 2.22 (s, 2H), and 2.18 (s, 3H). ^13^C-NMR (400 MHz, DMSO) δ 168.873, 161.152, 159.862, 158.730, 145.620, 136.764, 134.428, 131.993, 131.899, 130.622, 129.065, 128.365, 126.068, 124.370, 115.602, 54.709, 53.056, 52.491, 46.628, 41.219, 37.255, and 19.052 ppm.

4-(3-((4-(4-Ethylbenzoyl)piperazin-1-yl)methyl)-4-fluorobenzyl)phthalazin-1(2*H*)-one (**A10**). HRMS (AP-ESI) m/z calcd for C_29_H_29_FN_4_O_2_ [M + H]^+^ 485.23545, found 485.23273 [M + H]^+^. ^1^H-NMR (400 MHz, DMSO) δ 12.59 (s, 1H), 8.25 (dd, *J*_1_ = 8.0 Hz, *J*_2_ = 1.2 Hz, 1H), 7.91 (d, *J* = 8.0 Hz, 1H), 7.86–7.76 (m, 2H), 7.32–7.25 (m, 6H), 7.11–7.07 (m, 1H), 4.30 (s, 2H), 3.50 (s, 4H), 2.64 (q, *J* = 8.0 Hz, 2H), 2.23 (s, 4H), and 1.20 (t, *J* = 7.6 Hz, 3H). ^13^C-NMR (400 MHz, DMSO) δ 169.453, 161.164, 159.869, 158.754, 145.827, 134.435, 133.839, 133.622 (d, *J* = 21.8 Hz), 131.982, 131.914, 129.650, 129.559, 128.183 (d, *J* = 18.7 Hz), 127.587, 126.105, 124.525, 115.835, 54.711, 40.604, 40.396, 40.187, 37.258, 28.450, and 15.863 ppm.

4-(4-Fluoro-3-((4-(2-methoxybenzoyl)piperazin-1-yl)methyl)benzyl)phthalazin-1(2*H*)-one (**A11**). HRMS (AP-ESI) m/z calcd for C_28_H_27_FN_4_O_3_ [M + H]^+^ 487.21472, found 487.21201 [M + H]^+^. ^1^H-NMR (400 MHz, DMSO) δ 12.59 (s, 1H), 8.25 (q, *J* = 4.0 Hz, 1H), 7.91 (d, *J* = 4.0 Hz, 1H), 7.86–7.78 (m, 2H), 7.41–7.39 (m, 1H), 7.37–7.31 (m, 1H), 7.25–7.24 (m, 1H), 7.15–7.06 (m, 3H), 7.01–6.97 (m, 1H), 4.30 (s, 2H), 3.76 (s, 3H), 3.54 (s, 2H), 3.49 (s, 2H), 3.05 (s, 2H), and 2.36–2.33 (s, 4H). ^13^C-NMR (400 MHz, DMSO) δ 166.762, 161.153, 159.869, 158.733, 155.311, 145.623, 134.415, 133.812, 131.938 (d, *J* = 9.2 Hz), 131.892, 130.776, 129.557, 128.114, 126.035 (d, *J* = 4.7 Hz), 124.379 (d, *J* = 15.0 Hz), 121.075, 115.806 (d, *J* = 22.3 Hz), 111.770, 55.833, 54.738, 52.919, 52.425, 46.617, 41.365, and 37.260 ppm.

4-(3-((4-Benzoylpiperazin-1-yl)methyl)-4-fluorobenzyl)phthalazin-1(2*H*)-one (**A12**). HRMS (AP-ESI) m/z calcd for C_27_H_25_FN_4_O_2_ [M + H]^+^ 457.20415, found 457.20154 [M + H]^+^. ^1^H-NMR (400 MHz, DMSO) δ 12.59 (s, 1H), 8.25 (q, *J* = 4.0 Hz, 1H), 7.91 (d, *J* = 8.0 Hz, 1H), 7.86–7.76 (m, 2H), 7.46–7.43 (m, 3H), 7.37–7.23 (m, 4H), 7.19 (dd, *J*_1_ = 8.0 Hz, *J*_2_ = 1.2 Hz, 1H), 4.30 (s, 2H), 3.54 (s, 2H), 3.50 (s, 2H), 3.25 (s, 2H), and 2.30 (s, 4H). ^13^C-NMR (400 MHz, DMSO) δ 169.328, 161.162, 159.868, 158.743, 145.628, 136.319, 134.435 (d, *J* = 3.1 Hz), 133.824, 132.016, 131.907, 129.963, 129.562, 128.370, 127.355, 126.096, 124.519 (d, *J* = 15.1 Hz), 124.369, 115.832, 115.611, 54.697, 52.919, 52.431, 47.499, 41.919, and 37.260 ppm.

4-(4-Fluoro-3-((4-(4-methoxybenzoyl)piperazin-1-yl)methyl)benzyl)phthalazin-1(2*H*)-one (**A13**). HRMS (AP-ESI) m/z calcd for C_28_H_27_FN_4_O_3_ [M + H]^+^ 487.21472, found 487.21152 [M + H]^+^. ^1^H-NMR (400 MHz, DMSO) δ 12.59 (s, 1H), 8.25 (dd, *J*_1_ = 8.0 Hz, *J*_2_ = 1.2 Hz, 1H), 7.92–7.91 (m, 1H), 7.87–7.77 (m, 2H), 7.35–7.31 (m, 3H), 7.28–7.24 (m, 1H), 7.11–7.07 (m, 1H), 7.00–6.96 (m, 2H), 4.30 (s, 2H), 3.80 (s, 3H), 3.50 (s, 2H), 3.41 (s, 4H), and 2.32 (s, 4H). ^13^C-NMR (400 MHz, DMSO) δ 169.306, 161.162, 160.637, 159.891, 158.743, 145.652, 134.412, 133.829, 132.008, 131.910, 129.712, 129.482, 128.174 (d, *J* = 18.2 Hz), 126.075, 124.365 (d, *J* = 15.0 Hz), 115.820, 114.081, 55.695, 54.700, 52.704, 40.573, 40.156, 39.947, and 37.262 ppm.

1-(2-Fluoro-5-((4-oxo-3,4-dihydrophthalazin-1-yl)methyl)benzyl)piperidine-4-carboxamide (**A14**). HRMS (AP-ESI) m/z calcd for C_22_H_23_FN_4_O_2_ [M + H]^+^ 395.18850, found 395.18613 [M + H]^+^. ^1^H-NMR (400 MHz, DMSO) δ 12.59 (s, 1H), 8.25 (dd, *J*_1_ = 8.0 Hz, *J*_2_ = 1.2 Hz, 1H), 7.93 (dd, *J*_1_ = 8.0 Hz, *J*_2_ = 1.2 Hz, 1H), 7.87–7.79 (m, 2H), 7.34 (q, *J* = 4.0 Hz, 1H), 7.24–7.21 (m, 2H), 7.18–7.04 (m, 1H), 6.71 (s, 1H), 4.30 (s, 2H), 3.42 (s, 2H), 2.75–2.72 (m, 2H), 2.09–1.97 (m, 1H), 1.90–1.85 (m, 2H), 1.62–1.59 (m, 2H), and 1.54–1.45 (m, 2H). ^13^C-NMR (400 MHz, DMSO) δ 176.952, 159.853, 145.706, 134.390, 133.812, 131.941, 131.880, 129.559, 129.416, 128.372, 126.488, 126.100, 125.228, 115.728, 115.499, 55.150, 52.968, 42.037, 40.395, 39.769, 37.335, and 28.899 ppm.

4-(3-((4-(Benzo[*d*][1,3]dioxol-5-ylmethyl)piperazin-1-yl)methyl)-4-fluorobenzyl)phthalazin-1(2*H*)-one (**A15**). HRMS (AP-ESI) m/z calcd for C_28_H_27_FN_4_O_3_ [M + H]^+^ 487.21472, found 487.21194 [M + H]^+^. ^1^H-NMR (400 MHz, DMSO) δ 12.58 (s, 1H), 8.26–8.24 (m, 1H), 7.91–7.90 (m, 1H), 7.89–7.77 (m, 2H), 7.35–7.31 (m, 1H), 7.29–7.21 (m, 1H), 7.09–7.05 (m, 1H), 6.85–6.81 (m, 2H), 6.73–6.71 (m, 1H), 5.98 (s, 2H), 4.29 (s, 2H), and 2.27 (s, 8H). ^13^C-NMR (400 MHz, DMSO) δ 161.092, 159.870, 158.674, 148, 147.638, 146.566, 134.372, 133.777, 132.493, 131.688, 129.567, 129.344, 128.391, 126.483, 124.938, 122.349, 115.735, 109.470, 108.262, 101.218, 62.153, 54.698, 52.795, and 37.308 ppm.

4-(3-([1,4′-Bipiperidin]-1′-ylmethyl)-4-fluorobenzyl)phthalazin-1(2*H*)-one (**A16**). HRMS (AP-ESI) m/z calcd for C_26_H_31_FN_4_O [M + H]^+^ 435.25616, found 435.25369 [M + H]^+^. ^1^H-NMR (400 MHz, DMSO) δ 12.59 (s, 1H), 8.26–8.24 (m, 1H), 7.91–7.79 (m, 3H), 7.28–7.21 (m, 2H), 7.09–7.04 (m, 1H), 4.29 (s, 2H), 3.40 (s, 2H), 2.73–2.70 (m, 2H), 2.39 (s, 4H), 2.08 (m, 1H), 1.83 (t, *J* = 12.0 Hz, 2H), 1.56 (d, *J* = 12.0 Hz, 2H), 1.46–1.45 (m, 4H), and 1.37–1.31 (m, 4H). ^13^C-NMR (400 MHz, DMSO) δ 159.856, 145.680, 134.406, 133.803, 131.919, 131.660, 129.571, 129.271, 128.383, 126.464, 126.133, 125.171, 115.709, 115.476, 62.293, 54.798, 53.044, 50.133, 40.608, 40.400 (d, *J* = 21.0 Hz), 39.983, 39.357, 37.308, 27.843, 26.556, and 25.074 ppm.

Methyl1-(2-fluoro-5-((4-oxo-3,4-dihydrophthalazin-1-yl)methyl)benzyl)piperidine-4-carboxylate (**A17**). HRMS (AP-ESI) m/z calcd for C_23_H_24_FN_3_O_3_ [M + H]^+^ 410.18907, found 410.18555 [M + H]^+^. ^1^H-NMR (400 MHz, DMSO) δ 12.59 (s, 1H), 8.26–8.24 (m, 1H), 7.93–7.91 (m, 1H), 7.87–7.79 (m, 2H), 7.33–7.30 (m, 1H), 7.24–7.21 (m, 1H), 7.09–7.05 (m, 1H), 4.30 (s, 2H), 3.59 (s, 3H), 3.43 (s, 2H), 2.68–2.65 (m, 2H), 2.26 (s, 1H), 1.95 (dd, *J*_1_ = 9.6 Hz, *J*_2_ = 8.8 Hz, 2H), 1.75–1.72 (m, 2H), and 1.52–1.47 (m, 2H). ^13^C-NMR (400 MHz, DMSO) δ 175.251, 159.856, 145.689, 134.432, 133.815, 131.938, 131.814, 129.570, 129.447, 128.374, 126.483, 126.111, 115.523, 54.998, 52.403, 51.858, 40.606 (d, *J* = 21.0 Hz), 40.188, 39.771, 39.354, 37.294, and 28.353 ppm.

4-(3-((4-Benzylpiperidin-1-yl)methyl)-4-fluorobenzyl)phthalazin-1(2*H*)-one (**A18**). HRMS (AP-ESI) m/z calcd for C_28_H_28_FN_3_O [M + H]^+^ 442.22964, found 442.22733 [M + H]^+^. ^1^H-NMR (400 MHz, DMSO) δ 12.59 (s, 1H), 8.26–8.24 (m, 1H), 7.90–7.89 (m, 1H), 7.88–7.77 (m, 2H), 7.30–7.14 (m, 7H), 7.08–7.03 (m, 1H), 4.29 (s, 2H), 3.39 (s, 2H), 2.66–2.63 (m, 2H), 2.45 (s, 2H), 1.82–1.79 (m, 2H), and 1.44–1.41 (m, 2H). ^13^C-NMR (400 MHz, DMSO) δ 159.882, 145.666, 140.753, 133.786, 131.897, 129.561, 129.442, 128.580, 128.397, 126.480, 126.208, 126.124, 115.732, 115.526, 65.386, 54.943, 53.305, 42.738, 37.326, and 32.086 ppm.

4-(4-Fluoro-3-((4-(hydroxymethyl)piperidin-1-yl)methyl)benzyl)phthalazin-1(2*H*)-one (**A19**). HRMS (AP-ESI) m/z calcd for C_22_H_24_FN_3_O_2_ [M + H]^+^ 382.19326, found 382.19049 [M + H]^+^. ^1^H-NMR (400 MHz, DMSO) δ 12.59 (s, 1H), 8.26–8.24 (m, 1H), 7.93–7.91 (m, 1H), 7.87–7.79 (m, 2H), 7.32–7.30 (m, 1H), 7.22–7.21 (m, 1H), 7.09–7.04 (m, 1H), 4.39 (t, *J* = 4.0 Hz, 1H), 4.29 (s, 2H), 3.34 (s, 2H), 3.21 (d, *J* = 9.6 Hz, 2H), 2.71 (d, *J* = 12.0 Hz, 2H), 1.85 (dd, *J*_1_ = 9.6 Hz, *J*_2_ = 1.2 Hz, 2H), 1.57–1.54 (m, 2H), 1.27 (s, 1H), and 1.05–1.01 (m, 2H). ^13^C-NMR (400 MHz, DMSO) δ 161.106, 159.865, 158.692, 145.724, 134.365 (d, *J* = 3.1 Hz), 133.804, 131.937, 131.772, 131.727, 129.244 (d, *J* = 8.3 Hz), 128.370, 126.482, 125.315 (d, *J* = 14.7 Hz), 115.477, 66.394, 55.220, 53.298, 40.587, 39.335, 38.679, 37.315, and 29.129 ppm.

4-(3-((4-(Benzo[*d*][1,3]dioxole-5-carbonyl)piperazin-1-yl)methyl)-4-fluorobenzyl)phthalazin-1(2*H*)-one (**A20**). HRMS (AP-ESI) m/z calcd for C_28_H_25_FN_4_O_4_ [M + H]^+^ 501.19398, found 501.19138 [M + H]^+^. ^1^H-NMR (400 MHz, DMSO) δ 12.59 (s, 1H), 8.25 (dd, *J*_1_ = 8.0 Hz, *J*_2_ = 1.2 Hz, 1H), 7.93–7.90 (m, 1H), 7.87–7.79 (m, 2H), 7.33–7.31 (m, 1H), 7.25 (s, 1H), 7.09–7.07 (m, 1H), 6.97–6.93 (m, 2H), 6.88–6.86 (m, 1H), 6.07 (s, 2H), 4.30 (s, 2H), 3.50 (s, 2H), 3.40 (m, 4H), and 2.32 (s, 4H). ^13^C-NMR (400 MHz, DMSO) δ 168.816, 161.157, 159.868, 158.737, 148.656, 147.567, 145.635, 134.432, 133.844, 132.001, 131.922, 129.715, 128.363, 126.101, 124.403 (d, *J* = 14.8 Hz), 121.828, 115.829, 115.609, 108.559, 108.185, 101.891, 54.703, 52.653, and 37.255 ppm.

4-(3-((4-(Cyclopropanecarbonyl)piperazin-1-yl)methyl)-4-fluorobenzyl)phthalazin-1(2*H*)-one (**A21**). HRMS (AP-ESI) m/z calcd for C_24_H_25_FN_4_O_4_ [M + H]^+^ 421.20415, found 421.20166 [M + H]^+^. ^1^H-NMR (400 MHz, DMSO) δ 12.59 (s, 1H), 8.27–8.24 (m, 1H), 7.94–7.92 (m, 1H), 7.88–7.79 (m, 2H), 7.36–7.33 (m, 1H), 7.28–7.23 (m, 1H), 7.12–7.09 (m, 1H), 4.30 (s, 2H), 3.60 (s, 2H), 3.50 (s, 2H), 3.41–3.36 (m, 2H), 2.34 (s, 2H), 2.25 (s, 2H), 1.94–1.91 (m, 1H), and .71–.67 (m, 4H). ^13^C-NMR (400 MHz, DMSO) δ 171.328, 161.164, 159.866, 158.744, 145.659, 134.466 (d, *J* = 3.1 Hz), 133.851, 132.010, 131.953 (d, *J* = 5.7 Hz), 129.561, 128.372, 126.097, 124.398 (d, *J* = 15.1 Hz), 115.821, 115.599, 54.708, 53.203, 52.490, 45.229, 41.942, 40.607, 37.271, 10.600, and 7.444 ppm.

4-(3-((4-(4,4-Difluorocyclohexane-1-carbonyl)piperazin-1-yl)methyl)-4-fluorobenzyl) phthalazin-1(2*H*)-one (**A22**). HRMS (AP-ESI) m/z calcd for C_27_H_29_F_3_N_4_O_2_ [M + H]^+^ 499.23226, found 499.22986 [M + H]^+^. ^1^H-NMR (400 MHz, DMSO) δ 12.59 (s, 1H), 8.27–8.25 (m, 1H), 7.93–7.91 (m, 1H), 7.88–7.80 (m, 2H), 7.34–7.32 (m, 1H), 7.28–7.24 (m, 1H), 7.12–7.07 (m, 1H), 4.30 (s, 2H), 3.50 (s, 2H), 3.45 (s, 2H), 3.38 (s, 2H), 2.75 (s, 1H), 2.33 (s, 2H), 2.24 (s, 2H), 2.03–2.02 (m, 2H), 2.00–1.81 (s, 2H), 1.71–1.67 (m, 2H), and 1.60–1.53 (m, 2H). ^13^C-NMR (400 MHz, DMSO) δ 173.714, 161.158, 159.867, 158.739, 145.644, 134.424, 133.849, 132.000, 131.932, 129.613 (d, *J* = 13.2 Hz), 129.562, 128.372, 126.102, 124.398 (d, *J* = 15.2 Hz), 115.814 (d, *J* = 22.3 Hz), 54.705, 53.350, 52.602, 45.142, 41.304, 40.600, 40.391, 40.183, 37.270, 29.581, 26.046, and 25.615 ppm.

4-(3-((4-(Cyclohexanecarbonyl)piperazin-1-yl)methyl)-4-fluorobenzyl)phthalazin-1(2*H*)-one (**A23**). HRMS (AP-ESI) m/z calcd for C_27_H_31_FN_4_O_2_ [M + H]^+^ 463.25110, found 463.24875 [M + H]^+^. ^1^H-NMR (400 MHz, DMSO) δ 12.59 (s, 1H), 8.27–8.25 (m, 1H), 7.92 (d, *J* = 8.0 Hz, 1H), 7.87 (dd, *J*_1_ = 8.0 Hz, *J*_2_ = 1.2 Hz, 2H), 7.83 (dd, *J*_1_ = 4.0 Hz, *J*_2_ = 1.2 Hz, 2H), 7.33–7.32 (m, 1H), 7.28–7.24 (m, 1H), 7.12–7.07 (m, 1H), 4.30 (s, 2H), 3.48 (s, 2H), 3.40 (s, 2H), 3.36 (s, 2H), 2.51 (s, 2H), 2.30 (s, 2H), 2.23 (s, 2H), 1.69 (s, 2H), 1.64 (s, 1H), 1.59 (d, *J* = 8.0 Hz, 2H), and 1.34 (s, 4H). ^13^C-NMR (400 MHz, DMSO) δ 172.500, 161.165, 159.868, 158.744, 145.650, 134.444, 133.857, 131.954, 129.726, 128.369, 126.495, 126.097, 124.384 (d, *J* = 15.1 Hz), 115.826, 115.604, 54.675, 53.327, 52.505, 45.178, 41.430, 37.270, 32.907, 32.676, 32.437, 26.017, and 25.919 (d, *J* = 9.8 Hz) ppm.

### Preparation of **2d** (see as follows)

2-Fluoro-5-((4-oxo-3,4-dihydrophthalazin-1-yl)methyl)benzohydrazide (**2d**). Compound **2a** (.50 g, 1.60 mmol) and hydrazine hydrate (.32 g, 8.00 mmol, 80%) were mixed in methanol. The mixture was then heated up to 80°C and stirred for 24 h. After cooling to room temperature, the desired compound **2d** (.42 g, 84%) was derived by crystallization as white powder. HRMS m/z: 313.10779 [M + H]^+^. ^1^H-NMR (400 MHz, DMSO) δ 12.60 (s, 1H), 9.48 (s, 1H), 8.26 (d, *J* = 8.0 Hz, 1H), 7.97 (d, *J* = 8.0 Hz, 1H), 7.91–7.80 (m, 2H), 7.51–7.44 (m, 2H), 7.20–7.17 (m, 1H), 4.50 (s, 2H), and 4.32 (s, 2H).

### Preparation of **B1** and its analogs: Derivatives **B2–B6** were prepared as described for **B1** (see as follows)

*N*′-ethyl-2-fluoro-5-((4-oxo-3,4-dihydrophthalazin-1-yl)methyl)benzohydrazide (**B1**). To a stirred solution of **2d** (0.31 g, 1.00 mmol) in MeOH, acetaldehyde (0.07 g, 1.50 mmol) was added and stirred at room temperature for 1 h. Then NaBH_4_ (0.08 g, 2.00 mmol) was added to the solution. After 4 h of stirring, the solvent was evaporated under vacuum, and water was added. The desired compound, **B1** (0.31 g, 91%), was derived by filtering. HRMS (AP-ESI) m/z calcd for C_18_H_17_FN_4_O_2_ [M + H]^+^ 341.14155, found 341.13947 [M + H]^+^. ^1^H-NMR (400 MHz, DMSO) δ 12.60 (s, 1H), 9.73 (d, *J* = 4.0 Hz, 1H), 8.26 (d, *J* = 8.0 Hz, 1H), 7.98 (d, *J* = 8.0 Hz, 1H), 7.91–7.81 (m, 2H), 7.52 (d, *J* = 8.0 Hz, 1H), 7.47–7.44 (m, 1H), 7.23–7.18 (m, 1H), 5.10–5.08 (m, 1H), 4.33 (s, 2H), 2.83–2.76 (s, 2H), and 1.01 (t, *J* = 8.0 Hz, 3H). ^13^C-NMR (400 MHz, DMSO) δ 163.109, 159.860, 159.510, 157.046, 145.394, 134.816, 134.783, 133.976, 132.010, 130.398, 128.355, 127, 126.521, 123.487, 116.483, 45.727, 36.889, and 13.451 ppm.

2-Fluoro-5-((4-oxo-3,4-dihydrophthalazin-1-yl)methyl)-*N*′-propylbenzohydrazide (**B2**). HRMS (AP-ESI) m/z calcd for C_19_H_19_FN_4_O_2_ [M + H]^+^ 355.15720, found 355.15543 [M + H]^+^. ^1^H-NMR (400 MHz, DMSO) δ 12.60 (s, 1H), 9.73 (d, *J* = 4.0 Hz, 1H), 8.26 (d, *J* = 8.0 Hz, 1H), 7.98 (d, *J* = 8.0 Hz, 1H), 7.90 (t, *J* = 8.0 Hz, 1H), 7.84–7.81 (m, 2H), 7.51 (d, *J* = 8.0 Hz, 1H), 7.45 (s, 1H), 7.22–7.17 (m, 1H), 5.11 (s, 1H), 4.32 (s, 2H), 2.73 (q, *J*_1_ = 8.0 Hz, 2H), 1.48–1.39 (m, 2H), and .88 (t, *J* = 8.0 Hz, 3H). ^13^C-NMR (400 MHz, DMSO) δ 163.073, 159.864, 159.515, 157.051, 145.389, 134.808 (d, *J* = 3.4 Hz), 134.775, 133.969, 132.927(d, *J* = 8.3 Hz), 130.386 (d, *J* = 2.9 Hz), 128.354, 126.519, 123.487 (d, *J* = 15.7 Hz), 116, 53.287, 36.893, 21.174, and 12.048 ppm.

*N*′-butyl-2-fluoro-5-((4-oxo-3,4-dihydrophthalazin-1-yl)methyl)benzohydrazide (**B3**). HRMS (AP-ESI) m/z calcd for C_20_H_21_FN_4_O_2_ [M + H]^+^ 369.17285, found 369.17093 [M + H]^+^. ^1^H-NMR (400 MHz, DMSO) δ 12.60 (s, 1H), 9.73 (s, 1H), 8.26 (d, *J* = 8.0 Hz, 1H), 7.97 (d, *J* = 8.0 Hz, 1H), 7.91–7.87 (m, 1H), 7.84–7.81 (m, 1H), 7.51–7.44 (m, 2H), 7.22–7.17 (m, 1H), 5.08 (s, 1H), 4.33 (s, 2H), 2.76–2.73 (m, 2H), 1.44–1.28 (m, 4H), and .88 (t, *J* = 8.0 Hz, 3H). ^13^C-NMR (400 MHz, DMSO) δ 163.074, 159.870, 159.523, 157.057, 145.384, 134.808 (d, *J* = 3.3 Hz), 134.775, 133.960, 132.854 (d, *J* = 8.3 Hz), 131.995, 130.378 (d, *J* = 2.8 Hz), 128.351, 126.518, 123.470 (d, *J* = 15.7 Hz), 116.704, 51.088, 36.901, 30.084, 20.203, and 14.353 ppm.

2-Fluoro-*N*′-isobutyl-5-((4-oxo-3,4-dihydrophthalazin-1-yl)methyl)benzohydrazide (**B4**). HRMS (AP-ESI) m/z calcd for C_20_H_21_FN_4_O_2_ [M + H]^+^ 369.17285, found 369.17078 [M + H]^+^. ^1^H-NMR (400 MHz, DMSO) δ 12.60 (s, 1H), 9.74 (s, 1H), 8.26 (d, *J* = 8.0 Hz, 1H), 7.97 (d, *J* = 8.0 Hz, 1H), 7.89 (t, *J* = 8.0 Hz, 1H), 7.83 (t, *J* = 8.0 Hz, 1H), 7.51–7.45 (m, 2H), 7.22–7.17 (m, 1H), 5.10 (s, 1H), 4.33 (s, 2H), 2.60–2.57 (m, 2H), 1.77–1.67 (m, 1H), and .90 (d, *J* = 8.0 Hz, 6H). ^13^C-NMR (400 MHz, DMSO) δ 163.067, 159.859, 159.509, 157.046, 145.392, 134.803, 134.770 (d, *J* = 3.4 Hz), 133.982, 132.846 (d, *J* = 8.3 Hz), 130.356 (d, *J* = 2.8 Hz), 128.352, 126.521, 126.009, 123.487 (d, *J* = 15.7 Hz), 116.707 (d, *J* = 22.5 Hz), 59.376, 36.885, 26.871, and 21.043 ppm.

2-Fluoro-5-((4-oxo-3,4-dihydrophthalazin-1-yl)methyl)-*N*′-pentylbenzohydrazide (**B5**). HRMS (AP-ESI) m/z calcd for C_21_H_23_FN_4_O_2_ [M + H]^+^ 383.18850, found 383.18677 [M + H]^+^. ^1^H-NMR (400 MHz, DMSO) δ 12.59 (s, 1H), 9.72 (d, *J* = 8.0 Hz, 1H), 8.27–8.25 (m, 1H), 7.98–7.96 (m, 1H), 7.91–7.81 (m, 2H), 7.52–7.43 (m, 2H), 7.20 (dd, *J*_1_ = 8.0 Hz, *J*_2_ = 1.6 Hz, 1H), 5.10–5.01 (m, 1H), 4.33 (s, 2H), 2.75 (dd, *J*_1_ = 6.8 Hz, *J*_2_ = 6.4 Hz, 2H), 1.44–1.41 (m, 2H), 1.30–1.29 (m, 4H), and .88 (d, *J* = 8.0 Hz, 3H). ^13^C-NMR (400 MHz, DMSO) δ 163.052, 159.851, 159.496, 157.032, 145.391, 134.814, 134.781, 133.987, 132.027, 130.380, 128.354, 126.523, 126.017, 123.337, 116.489, 51.361, 36.886, 29.234, 27.575, 22.497, and 14.382 ppm.

2-Fluoro-*N*′-hexyl-5-((4-oxo-3,4-dihydrophthalazin-1-yl)methyl)benzohydrazide (**B6**). HRMS (AP-ESI) m/z calcd for C_22_H_25_FN_4_O_2_ [M + H]^+^ 397.20415, found 397.20239 [M + H]^+^. ^1^H-NMR (400 MHz, DMSO) δ 12.59 (s, 1H), 9.72 (d, *J* = 8.0 Hz, 1H), 8.27–8.25 (m, 1H), 7.98–7.96 (m, 1H), 7.91–7.81 (m, 2H), 7.51–7.43 (m, 2H), 7.20 (dd, *J*_1_ = 8.0 Hz, *J*_2_ = 1.6 Hz, 1H), 5.10–5.05 (m, 1H), 4.33 (s, 2H), 2.77–2.72 (m, 2H), 1.44–1.34 (m, 2H), 1.32–1.22 (m, 6H), and .86 (d, *J* = 8.0 Hz, 3H). ^13^C-NMR (400 MHz, DMSO) δ 163.036, 159.849, 159.506, 157.041, 145.381, 134.807, 134.775 (d, *J* = 3.2 Hz), 133.975, 132.846 (d, *J* = 8.2 Hz), 130.364, 128.357, 126.521, 126.005, 123.326 (d, *J* = 15.6 Hz), 116.482, 51.413, 36.893, 31.672, 27.883, 26.714, 22.529, and 14.392 ppm.

### Preparation of **2e** (see as follows)

*Tert*-butyl 4-(2-fluoro-5-((4-oxo-3,4-dihydrophthalazin-1-yl)methyl)benzoyl)piperazine-1-carboxylate (**2e**). To the solution of compound **2** (.50 g, 1.68 mmol) in DCM, Et_3_N (.51 g, 5.04 mmol) and TBTU (.65 g, 2.02 mmol) were added at 0°C. The solution was kept at 0°C for 20 min, and then 1-Boc-piperazine (.38 g, 2.02 mmol) was added. The reaction mixture was stirred at room temperature overnight. The solvent was then evaporated, and the compound was dissolved in EtOAc (30 ml). The EtOAc solution was washed with 1 M citric acid (3 × 30 ml), NaHCO_3_ (3 × 30 ml), and brine (3 × 30 ml). The organic phase layer was dried over Mg_2_SO_4_ and concentrated *in vacuo*. The desired compound **2e** (.64 g, 78%) was derived by crystallization in EtOAc as white powder. HRMS m/z: 489.18884 [M + H]^+^. ^1^H-NMR (400 MHz, DMSO) δ 12.60 (s, 1H), 8.27 (d, *J* = 8.0 Hz, 1H), 7.97 (d, *J* = 8.0 Hz, 1H), 7.91–7.88 (m, 1H), 7.84 (t, *J* = 8.0 Hz, 1H), 7.46–7.43 (m, 1H), 7.36 (d, *J* = 4.0 Hz, 1H), 7.24 (t, *J* = 8.0 Hz, 1H), 4.33 (s, 2H), 3.59 (s, 2H), 3.38 (s, 2H), 3.23 (s, 2H), 3.15 (s, 2H), and 1.41 (s, 9H).

### Preparation of **2f** (see as follows)

4-(4-Fluoro-3-(piperazine-1-carbonyl)benzyl)phthalazin-1(2*H*)-one (**2f**). The solution of **2e** in DCM with TFA was stirred for 2 h. Then the solution was concentrated *in vacuo* to get **2f**, which was used for the next step reaction without purification.

### Preparation of **C1** and its analogs: Derivative **C2** was prepared as described for **C1** (see as follows)

4-(3-(4-(4-(Bis(2-chloroethyl)amino)benzoyl)piperazine-1-carbonyl)-4-fluorobenzyl) phthalazin-1(2*H*)-one (**C1**). To the solution of 4-[bis(2-chlorethyl)amino]benzoic acid (.27 g, 1.02 mmol) in DCM, Et_3_N (.31 g, 3.06 mmol) and TBTU (.39 g, 1.22 mmol) were added at 0°C. The solution was kept at 0°C for 20 min, and then **2f** (.47 g, 1.22 mmol) was added. The reaction mixture was stirred at room temperature overnight. The solvent was then evaporated, with the residue taken up in EtOAc (30 ml) and then washed with 1 M citric acid solution (3 × 30 ml), saturated NaHCO_3_ solution (3 × 30 ml), and saturated brine solution (3 × 30 ml). The organic phase layer was dried over Mg_2_SO_4_ and concentrated *in vacuo*. The resulting residue was purified by flash column chromatography (PE:EA, 1: 4 v/v) to obtain the pure product **C1** (0.39 g, 63%) as white powder. HRMS (AP-ESI) m/z calcd for C_31_H_30_Cl_2_FN_5_O_3_ [M + H]^+^ 610.17897, found 610.17621 [M + H]^+^. ^1^H-NMR (400 MHz, DMSO) δ 12.59 (s, 1H), 8.26 (d, *J* = 8.0 Hz, 1H), 7.96 (d, *J* = 8.0 Hz, 1H), 7.88 (t, *J* = 8.0 Hz, 1H), 7.81 (t, *J* = 8.0 Hz, 1H), 7.44 (s, 1H), 7.36 (d, *J* = 4.0 Hz, 1H), 7.31 (d, *J* = 8.0 Hz, 2H), 7.24 (t, *J* = 8.0 Hz, 1H), 6.78 (d, *J* = 8.0 Hz, 2H), 4.33 (s, 2H), 3.76 (s, 8H), 3.65 (s, 2H), 3.57 (s, 2H), 3.42 (s, 2H), and 3.22 (s, 2H). ^13^C-NMR (400 MHz, DMSO) δ 170.047, 164.508, 159.855, 148.180, 145.315, 135.295, 133.952, 132.016, 130.018, 129.550, 128.366, 126.544, 125.946, 123.928, 123.180, 111.422, 52.312, 41.420, 40.609, 40.400, 40.192, 39.983, 39.775, 39.358, and 36.901 ppm.

4-(3-(4-(4-(4-(Bis(2-chloroethyl)amino)phenyl)butanoyl)piperazine-1-carbonyl)-4-fluorobenzyl)phthalazin-1(2H)-one (**C2**). HRMS (AP-ESI) m/z calcd for C_34_H_36_Cl_2_FN_5_O_3_ [M-H]^-^ 650.20992, found 650.21179 [M-H]^-^. ^1^H-NMR (400 MHz, DMSO) δ 12.59 (s, 1H), 8.26 (dd, *J* = 8.0, 1.2 Hz, 1H), 7.96 (d, *J* = 8.0 Hz, 1H), 7.90 (t, *J* = 7.8, 1.5 Hz, Hz, 1H), 7.87–7.80 (m, 1H), 7.44 (s, 1H), 7.36 (t, *J* = 3.0 Hz, 1H), 7.24 (t, *J* = 8.0 Hz, 1H), 7.03 (d, *J* = 4.0 Hz, 2H), 6.67 (q, *J* = 4.0 Hz, 2H), 4.33 (s, 2H), 3.69 (d, *J* = 4.0 Hz, 8H), 3.62–3.51 (m, 4H), 3.37 (s, 2H), 3.15 (s, 2H), 2.47 (d, *J* = 4.0 Hz, 2H), 2.34 (t, *J* = 8.0 Hz, 1H), 2.27 (t, *J* = 6.9 Hz, 1H), and 1.73 (d, *J* = 8.0 Hz, 2H). ^13^C-NMR (400 MHz, DMSO) δ 171.126, 164.441, 159.859, 155.612, 145.302, 144.866, 135.278, 133.954, 132.180 (d, *J* = 15.8 Hz), 130.415, 129.769, 129.553, 129.395 (d, *J* = 15.9 Hz), 128.374, 126.549, 124.133, 116.508, 116.295, 112.351, 52.673, 47.099, 45.361, 44.946, 42.092, 41.609, 40.611, 40.194, 36.908, 34.042, 32.154, and 27.240 ppm.

### *In vitro* antiproliferative assay

The proliferation of cancer cells was tested by CCK-8 assay. Briefly, cells were seeded in 96-well plates at approximately 5 × 10^3^ cells per well. The cells were treated with test compounds after 24 h of incubation. CCK-8 reagent (10 ml) was added to each well after 72 h of incubation, and the cells were then incubated at 37°C for 4 h. The light absorbance at 450 nm was measured using an Opsys microplate reader (Dynex Technologies, Chantilly, VA, United States). Results are illustrated as percentage viability normalized to DMSO-treated control cells.

### Cell cycle analysis

K562 and HCC1937 cells were incubated with different doses of molecule **C2**, olaparib, or chlorambucil for 24 h. After treatment, cells were collected and fixed with 70% pre-chilled ethanol in PBS and stored at −20°C overnight. The cells were then washed twice with PBS, incubated with 100 μg/ml RNase I (Solarbio, China) at 37°C for 1 h, and stained with propidium iodide (PI, 10 μg/ml, Solarbio, China) for 30 min, in the dark, at room temperature. Finally, the DNA content was measured using flow cytometry (FACSAria III, Becton Dickinson, United States). The data were analyzed and fitted using ModFit software.

### Cell apoptosis analysis

K562 cells were treated with various concentrations of molecule **C2**, olaparib, or chlorambucil for 24 h. Cells were then harvested and washed twice with PBS and then resuspended with binding buffer (Becton Dickinson, United States). Cells were incubated with Annexin V-BV421 (Becton Dickinson, United States) and 7-AAD (Becton Dickinson, United States) for double labeling for 30 min, in the dark, at room temperature, followed by analysis using flow cytometry (FACSAria III, Becton Dickinson, United States). The data were analyzed using FlowJo v10 software.

## Data Availability

The original contributions presented in the study are included in the article/supplementary material; further inquiries can be directed to the corresponding authors.
